# Bioactive metabolites from endophytic *Fusarium equiseti* isolated from *Hyoscyamus muticus* mitigate cadmium toxicity in biological models

**DOI:** 10.1038/s41598-026-59146-x

**Published:** 2026-06-30

**Authors:** Hagar E. Mohammed, Mervat G. Hassan, Ahmed A. Hamed, Monga I. Mossa

**Affiliations:** 1 Zoology Dep., Faculty of Science, Arish University, Arish, 45511 North Sinai Egypt; 2 Department of Botany and Microbiology, Faculty of Science, Benha University, Benha, Egypt; 3 Microbial Chemistry Department, National Research Center, Giza, Egypt; 4 Department of Botany and Microbiology, Faculty of Science, Arish University, Arish, North Sinai Egypt

**Keywords:** Cadmium chloride, *Fusarium equiseti*, Bioactive secondary metabolites, Endophytic fungi, *Hyoscyamus muticus*, Oxidative stress, *In Silico*, Biochemistry, Drug discovery, Microbiology, Plant sciences

## Abstract

Cadmium is a hazardous metal that induces oxidative stress, hepato-renal dysfunction, and metabolic disturbances. This study investigated the protective potential of bioactive secondary metabolites extracted from endophytic *Fusarium equiseti* (FE) against cadmium-induced toxicity. Gas chromatography- Mass Spectrometry (GC–MS) profiling revealed 17 compounds dominated by esters (67%) and fatty acids (26%). The major constituents were 9,12-Octadecadienoic acid (Z,Z) (10.21%), and Oleic acid (8.13%). *In vitro*-FE exhibited dose dependent antioxidant activity (IC50 = 145.91µ g/ml in DPPH assay) and broad-spectrum antimicrobial efficacy, producing inhibition zones up to 46 mm against *E. coli*. Anti- inflammatory evaluation showed hemolysis inhibition rising from 3.1% (100 µg/mL) to 55.9% (1000 µg/ml). Furthermore, the MTT assay revealed that the FE exhibited moderate cytotoxicity against HeLa and PC3 (IC50 values of 194.15 µg/ml and 162.88 µg/ml, respectively) cancer cell lines, while showing a lower toxicity toward normal WI38 cells (IC50 of 237.26 µg/ml; Selectivity Index up to 1.46). These metabolites are suggested to be the key contributors to the extract’s antioxidant and antimicrobial activities. *In vivo*, twenty Wistar rats were randomly assigned to four sets (n = 5 per set): (1) Control; (2) CdCl_2_, receiving a single IP dose of 3 mg/kg; (3) FE (100 mg/ kg; OP; for 14 constitutive days; and (4) FE + CdCl_2._ CdCl_2_ significantly elevated WBCs, lymphocyte, MDA, ALT, AST, ALP, creatinine, uric acid, total lipids, TG, CH, and LDL- C while reducing bilirubin, albumin, HDL-C, SOD, and GSH. Pretreatment with FE markedly restored antioxidant markers (SOD and GSH), reduced MDA, normalized lipid and liver profiles, and improved hepato-renal histopathology. These findings show that FE metabolites exert antioxidant, antimicrobial, anti-inflammatory, and cytoprotective effects, mitigating cadmium- induced oxidative damage. FE represents a promising natural source for developing hepato-renal protective agents against heavy metal toxicity.

**Supplementary Information:**

The online version contains supplementary material available at 10.1038/s41598-026-59146-x.

## Introduction

Among the world’s most important reactive hazardous metals, cadmium (Cd) has seen a dramatic rise in environmental concentrations from both natural and anthropogenic sources . Several possible pathways exist in the biosphere which could enter the human body. These include industrial and manufacturing processes, as well as the pollution of food, water, and air ^1^. Another major way to get Cd is to smoke. Organs such as the kidneys, liver, brain, lungs, heart, and skeletal system are severely affected by the metal’s multitarget, cumulative toxicant effects, which are mostly brought into the body by inhalation or consumption, in both animals and humans ^2^. Although the liver usually metabolizes highly toxic chemicals into non-toxic substances, it can also activate the less toxic metabolites into more toxic ones. Therefore, chemical burdens increase the stress on liver cells, leading to liver diseases such as cell degeneration, necrosis, and tumors ^3^. The hepatotoxicity of cadmium chloride (CdCl_2_) has been extensively studied in rats, with several studies showing that it causes cell necrosis and lipid alterations ^4^. The leakage of hepatic enzymes into the bloodstream is a direct consequence of cellular necrosis. Cd accumulation within the renal glomeruli and proximal tubules induces nephrotoxicity, rendering the kidney the most susceptible organ to chronic Cd exposure ^5^. When it comes to excretion, kidneys are among the most crucial organs. Hormones not only regulate blood pressure and keep fluid and electrolyte levels in the body in a steady state, but they may also alter the kidney’s structure and function by interacting with its arteries, glomeruli, and tubules ^6^.

Researchers are fighting a variety of diseases that are affected by environmental and lifestyle changes. Many scientists are working to better understand these emerging diseases and develop treatments using different chemical compositions and natural preparations. As reported by Rizvi *et al*.^7^, number of fungal endophyte species are being studied for their bioactive chemicals, which might have medicinal, agricultural, or industrial uses.

Endophytic fungi are microscopic organisms that spend most or all of their life cycles within plant tissues, where they do not create any outward signs of illness or harm ^8^. Extensive research has demonstrated that endophytes play a protective role in host plants by defending against harmful insects and diseases. On the other hand, endophytic fungi possess the remarkable ability to mimic the bioactive substances produced via their host plants, including flavonoids, coumarins, alkaloids, lignans, terpenoids, quinones, phenylpropanoids, glycosides, saponins, and xanthones^9^. Additionally, secondary metabolites from fungal endophytes have shown therapeutic benefits, including antioxidants, anti-cholesterol, anticancer, antibacterial, and antidiabetic activities ^10^. The North Sinai ecosystem is home to a community of approximately 100–120 medicinal plant species, including *Hyoscyamus muticus* L. (*H*. *muticus* L). In the wild, many fungal species inhabit *H*. *muticus* L. plants ^11^.

Furthermore, in nature, there are several endophytic fungi that are hosted on the *H*. *muticus* plant, with demonstrated potential to suppress diverse phytopathogen, as well as the plant itself, may synthesize a broad spectrum of fungi metabolites, such as alkaloids, and many more ^12^. Native to Egypt’s dry zone, the Solanaceae-family plant *H. muticus* is locally known as corn henna. It contains many phytochemicals with medicinal uses, including tropane alkaloids and hyoscyamine, that influence the brain and spinal cord ^13^.

It is reasonable to assume that *H. muticus* hosts endophytic fungi that can biosynthesize active chemicals, given that these fungi capable of biosynthesizing active molecules that are comparable to those of their host plants. Cancer, oxidative stress-related disorders, malaria, and inflammatory conditions are only a few of the many conditions that have benefited from the compounds produced by the endophytic fungus genera Fusarium and Penicillium ^14^. Fusarium is a huge genus found all over the world. Its seventy-plus species may colonize many different types of plants and generate a diverse array of active metabolites ^15^. Endophytic fungi, those belonging to the genus Fusarium, are recognized as prolific sources of bioactive secondary metabolites with diverse pharmacological applications. Among these, *Fusarium equiseti* (*F. equiseti*) has gained significant attention due to its ability to synthesize compounds with potent antioxidant, antimicrobial, and anti-inflammatory properties ^16,17^. Previous studies have highlighted its role in producing unique chemical scaffolds, such as fatty acids and phenolic derivatives, which exhibit selective cytotoxicity against various cancer cell lines and contribute protection against heavy metal-induced oxidative damage ^18,19^. In the context of environmental toxicology, *F. equiseti* metabolites have shown promise in modulating cellular defense mechanisms, making it a viable candidate for developing natural protective agents against chemically induced hepato-renal dysfunction ^19,20^.

Thus, the primary goal of this research was to determine whether or not ethyl acetate extract of *Fusarium equiseti* secondary metabolites (FE), which are derived from *H. muticus*, had any protective effect against cadmium chloride toxicity and its biological activities.

## Materials and methods

Cadmium chloride (CdCl_2_) was purchased from Sigma Co., USA and prepared in saline. Isoflurane, ethanol and ethyl acetate were sourced from Adwic-El Nasr Pharmaceutical Co. (Cairo, Egypt). Biochemical analyses were performed using assay kits from Bio-diagnostic Egypt, Spinreact (Girona, Spain), BIOMED Diagnostics (Oberschleissheim, Germany), Diamond Diagnostics (Germany), and SPECTRUM (Egypt). Potato dextrose agar (PDA) was bought from Merck Chemical Co. in Darmstadt, Germany. All other chemicals and reagents used were of analytical quality and were obtained from a standard commercial source.

### Plant samples collection

Wild specimens of *H*. *muticus* were collected from Al Hassana region of North Sinai Desert (30.5260°N. The collected plant material was properly labelled, transported to the microbiology laboratory at faculty of science, Arish University, where they were refrigerated until used for endophytic fungal isolation. Taxonomic identification was confirmed by a taxonomist, Dr. Nashwa A. M. Mostafa and a voucher specimen was deposited at the Herbarium of the Faculty of Science, Arish University, North Sinai, Egypt, under deposition number SO224.

All research and field studies on wild plants in our study, including the collection of plant material, fully comply with relevant institutional, national (Egyptian Ministry of Agriculture and Land Reclamation permits), and international guidelines and legislation. We have adhered to the IUCN Policy Statement on Research Involving Species at Risk of Extinction (1989) and the Convention on the Trade in Endangered Species of Wild Fauna and Flora (CITES); none of the studied species are listed as threatened, and all collections were conducted with prior ethical approval from our institutional review board and local authorities.

### Isolation of fungal secondary metabolites from wild *Hyoscyamus muticus*

Secondary metabolites of fungi were isolated from *H. muticus* following the protocols described by Kumar and Kaushik ^21^. Fresh, healthy lateral shoots of *H. muticus* were repeatedly washed with sterile distilled water (SDW) to eliminate surface debris. The shoots were then left to drain for 1 h to eliminate the preserving water. Surface sterilization was carried out by subsequent washing with SDW and then submerged in 70% ethanol for one minute. After a further washing in SDW, the parts were submerged in 0.5% sodium hypochlorite for one minute. Following this, they were rinsed three times with SDW. Healthy sterilized lateral shoots were cut into 1 cm^2^ pieces and dried on Whatman No.1 filter paper No.1 under laminar airflow. Then dried segments were placed on prepared plates of potato dextrose agar (PDA) using 200 g of potato, 20 g dextrose and 20 g of agar, and incorporated streptomycin (100 mg/l), and chloramphenicol (250 mg/l) to inhibit bacterial growth. Four slices were positioned on one side of each plate and maintained at room temperature until fungal growth was observed. 4–5 drops of final rinse water were inoculated onto PDA plates and incubated for 5–6 days to verify the efficacy of surface sterilization. Fungal colonies development was monitored daily, and emerging fungal colonies were cultured onto fresh PDA plate then kept at 4 °C on PDA slants. The microscopic features and colony shape of fungal isolates served as a basis for their identification.

### Production of bioactive compounds

**Fig. 1:**
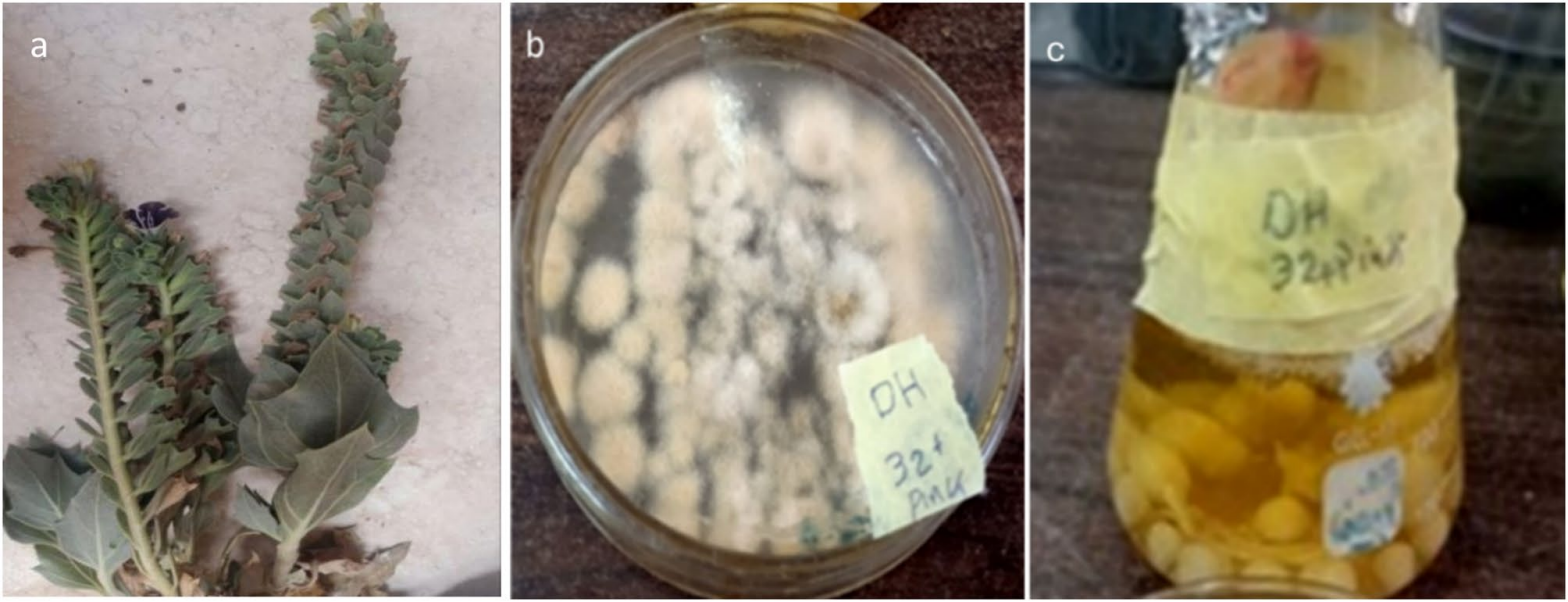
(**a**) *Hyoscyamus muticus* fresh plant, (**b**) Isolated fungal strain AUMC, and (**c**) Fermentation and extraction step.

Malt based nutrient broth (200 ml medium/250 ml Erlenmeyer flask) was prepared according to Magdi *et al*.^22^. Endophytic fungus *Fusarium equiseti* was selected to study its antibacterial and antifungal effects. Placing each flask under sterile conditions, three (8 cm) disks of each fungus were injected, the inoculated flasks were placed on a rotary shaker set at 180 rpm at 30 ℃ for 10 days. The incubation process was followed by the addition of 50 ml of ethyl acetate to each flask. Metabolites extraction was carried out twice at 40°C for 1.5 h at 120 rpm. After that, the upper organic phase was collected and concentrated by rotary evaporator (BUCHI). Before being used again, the concentrated sample was resuspended in pH 7.4 phosphate-buffered saline (Fig. 1c).

### Molecular identification of fungal isolates

Potato Dextrose agar (PDA) medium was used to grow the fungal isolates, which were then incubated at 28 °C for 5 days ^23^. Within the Molecular Biology Research facility of Assiut University, DNA was extracted using a kit sold in Korea by Intron Biotechnology called Patho-gene-spin DNA/RNA extraction. Daejeon, South Korean-based business SolGent helped us with the polymerase chain reaction (PCR) and sequence analysis. The internal transcribed spacer (ITS) region of the rRNA of fungal isolate was amplified using the primers ITS1 (forward: 5’—TCCGTAGGTGAA CCTGCGG—3’) and ITS4 (reverse:5’—TCCTCCGCTTATTGATATGC -3’) were used for identification of the rRNA of fungal isolate. The purified PCR products were subsequently sequenced using the same primers, and ddNTPs were added to the reaction mixture ^24^. The results of the sequence analysis were evaluated using the Basic Local Alignment Search Tool (BLAST), which may be found on the NCBI website. When analyzing sequences and building the phylogenetic tree, the program MegAlign software (DNASTAR,version 5.05) was employed.

### Preparation of endophytic fungal metabolites extract

The dried fungal extract was resuspended in pH 7.4 phosphate-buffered saline for studying its biological activity and i*n vivo* study.

### Gas chromatography–mass spectrometry (GC–MS) analysis

Chemical profiling was conducted using a Trace GC1310–ISQ mass spectrometer (Thermo Scientific, Austin, TX, USA) equipped with a TG–5MS capillary column (30 m × 0.25 mm × 0.25 µm). The oven temperature was programmed from 50 °C (initial hold) to 230 °C at 5 °C/min (2 min hold), then ramped to 290 °C at 30 °C/min (2 min hold). Injector and transfer line temperatures were maintained at 250 °C and 260 °C, respectively. Helium served as the carrier gas at 1 mL/min. A 1 µL sample was injected in split mode using an AS1300 autosampler after a 3 min solvent delay. Electron ionization spectra were acquired at 70 eV over m/z 40–1000 in full scan mode, with the ion source at 200 °C. Compound identification was based on retention times and mass spectra compared against NIST 11^25^ and WILEY 09 ^26^, libraries.

### Biological assays of the *Fusarium equiseti* secondary metabolites ethyl acetate extract (FE)

#### Antioxidant capacity

The antioxidant potential of FE extract was assessed using the 1, 1- diphenyl-2-picryl hydrazyl (DPPH) radical scavenging assay, as outlined by Baliyan *et al*.^27^. 0.1 mM DPPH solution in ethanol was prepared in ethanol, and 1 ml of the solution was mixed with 3 ml of extract solutions at graded concentrations (1.95, 3.9, 7.8, 15.62, 31.25, 62.5, 125, 250, 500, 1000 μg/ml). Only ethanol soluble fractions were used, and serial dilutions were performed to achieve the desired concentration. The reaction mixtures were vigorously shaken and incubated at ambient temp for 30 min. Absorbance was recorded at 517 nm using a Milton Roy UV–VIS spectrophotometer. Ascorbic acid served as positive control and all assays were done in triplicate. The DPPH radical scavenging activity percentage was estimated using the following expression:

$$Scavenging effect (\%) = \frac{A0-A 1 }{A0} \times 100$$

where A0 represents the control absorbance and A1 denotes the absorbance in presence of the test or standard sample.

The IC50 value, representing the concentration required to inhibit 50% of the DPPH radicals, was determined from the Log dose inhibition curve. A lower absorbance indicated higher free radical scavenging activity.

#### Antimicrobial efficacy

The antibacterial potential of the FE extract was measured using the agar well diffusion technique at The Regional center of Mycology and Biotechnology (RCMB), Al-Azhar University. The following microorganisms were cultivated according to the guidelines provided and adjusted to a concentration of 0.5 McFarland standard: *Staphylococcus aureus* ATCC25923, *Bacillus subtilis*, *Escherichia coli* ATCC25922, *Candida albicans* (ATCC 10221), *Pseudomonas aeruginosa*, and *Aspergillus fumigatus *. A sterile cotton swab was used to inject equal quantities into two different types of agar media: one for bacteria, Mueller–Hinton agar, and another, Sabouraud dextrose agar for fungi. Well, with a diameter of 6 mm were aseptically prepared using a sterile cork-borer, and 100 µl L of extract was carefully poured into each well. The positive control was gentamicin (4  µg/mL) for bacteria and ketoconazole (100 mg/mL) for fungi. The incubation temperatures for the bacteria and fungus were 37 degrees for 24 h and 28 degrees for 48 h, respectively, and the inhibitory zones were measured in millimeters^28^.

#### Anti-inflammatory activity

A modified version of the procedure reported by Asgary *et al*.^29^ was used to determine the anti-inflammatory activity. A quick rundown: once blood was drawn, it was spun in a centrifuge to resuspend the red blood cells in an isotonic buffer. After being treated with red blood cells for 1 h at 37°C, extracts in hypotonic and isotonic solutions (ranging from 100–1000 μg/ml) were then centrifuged. Hemolysis was assessed at 540 nm, and inhibition was calculated using $\% inhibition = 1-\frac{OD2-OD1}{OD30-OD1} \times 100$.

Where OD1 represents the absorbance of the test sample in isotonic solution.OD2, denotes the absorbance of the test sample in hypotonic solution, and OD3, corresponds to the absorbance of the control sample in hypotonic solution.

#### *In Vitro* cytotoxicity

The MTT protocol was used to assess cytotoxic activity in accordance with the methodology of Lara-Hernández *et al*.^30^. To summarize, a monolayer of cells was achieved by seeding them onto 96-well plates and then incubating them. After washing, serial dilutions of the test samples were added, while the control group received only maintenance medium. After incubation, toxicity was evaluated from morphological changes. MTT solution was added, mixed, and incubated until metabolic stimulation. After removing the medium, formazan was dissolved in DMSO, and the absorbance was measured at 560 nm nm was recorded. The absorbance background at 620 nm was subtracted in relation to cell viability. This cell line has been widely utilized in food safety and toxicology research, including studies on phytochemicals and plant growth regulators, with provenance documented in numerous peer-reviewed publications. As an established commercial cell line, no additional ethical approvals were required beyond ATCC’s standard certification of origin and quality control, which confirms ethical sourcing compliance with international guidelines such as those from the International Society for Stem Cell Research (ISSCR).

### Selectivity Index (SI) of FE extract

The calculation of Selectivity Index (SI) was determined by the following formula:

$$\frac{\mathrm{I}\mathrm{C}50 \mathrm{o}\mathrm{f} \mathrm{W}\mathrm{i}38}{\mathrm{I}\mathrm{C}50 \mathrm{o}\mathrm{f} \mathrm{c}\mathrm{a}\mathrm{n}\mathrm{c}\mathrm{e}\mathrm{r} \mathrm{c}\mathrm{e}\mathrm{l}\mathrm{l} \mathrm{l}\mathrm{i}\mathrm{n}\mathrm{e}}$$

#### Animals

The adult male *albino* rats used in this study were procured from VACERA, a Cairo, Egypt-based vaccine company. Prior to the experiment, the rats spent one week housed and acclimated in the laboratory’s Animal House at Arish University’s Faculty of Science in El Arish, Egypt. The animals were housed in a well-ventilated room with normal temperature regulation (22–24°C) and maintained on a 12-h dark/light cycle. All rats had unrestricted access to water and were fed rat pellets. Arish University Faculty of Science’s Animal Ethical clearance for this work (No. ARU006) was obtained by the Animal Ethics Committee of the Arish University College of Science. The entire research process complied with the ARRIVE guidelines and standard outlined in 1985 by the National Institutes of Health (NIH) publication, the Guide for the Care and Use of Laboratory Animals.

#### Study design

The study utilized a total of twenty male *albino* rats, aged approximately two to three months and weighting between 150 to 200 g. Randomly, the rats were distributed evenly into 4 groups of five rats each for 14-day administration period. The control group (G1) administered an intraperitoneal (IP) injection of saline for 14 days. The cadmium chloride group (G2) received CdCl₂ in a single IP dose of 3 mg/kg of body weight^31^. The FE group (G3) administered fungal Fusarium extract (FE) orally (PO) at a dose of 100mg/kg/day^32^ for 14 consecutive days. Finally, the FE + CdCl₂ group (G4) received FE at 100mg/kg/day, PO for 14 days, followed by a single IP injection of CdCl_2_ (3 mg/kg). The dosage was selected based on previous literature.

During the administration course, the animals were closely monitored for any signs of abnormalities, and their body weights were recorded at the conclusion of the study.

#### Blood sampling and organ harvesting

After 24 h of CdCl_2_ injection, the animals were deprived of water overnight. Anesthesia was induced using isoflurane, after which the neck hair was plucked and decapitated. The jugular vein was then gently severed using a sterile scalpel. Blood samples intended for hematological examination were collected in tubes containing the anticoagulant ethylenediaminetetraacetic acid (EDTA). For biochemical assessments, separate blood samples were collected and allowed to coagulate. Once clot formation was complete, the samples were centrifuged at 3,000 rpm for 20 min. to separate the serum, which was subsequently stored at -80°C until further analysis. The kidney and liver were also collected for assessing the levels of oxidative stress markers and for histological examinations.

#### Determination of body and relative organ weight

By keeping track of each group’s starting and ending weights during the 14-day trial, we were able to determine their body weight (BW). The animals were dissected and their organs—liver, kidney, heart, testes, and spleen—were promptly removed. Following a thorough cleaning with saline, the organs were prepared for further processing. . To get the relative weight, the total weight of the organs was partitioned by the total weight of the body (× 100).

#### Determination of hematological parameters

Following the manufacturer’s instructions, a Horiba ABX 80 Diagnostics system (ABX Pentra Montpellier, France) was used to examine fresh EDTA blood samples. The following tests were run: Red blood cell count (RBCs), white blood cell count (WBC), lymphocytes (LYM), hemoglobin content (HB), mean corpuscular hemoglobin (MCH), mean corpuscular hemoglobin concentration (MCHC), mean corpuscular volume (MCV), and platelet count (PLT).

### Biochemistry parameters analysis

#### Liver functions determination

Reference: GL-13–20, a kit from Bio-diagnostic Egypt was used to assess serum glucose level. Furthermore, a commercial kit from Spinreact (Girona, Spain) was used to evaluate the activities of serum alanine (ALT) and aspartate (AST) aminotransferases^33^. A commercial kit from BIOMED Diagnostics, Oberschleißheim, Germany (REF: TP116150), was used to assess the total protein concentration^34^. Diamond Diagnostics (Germany) kits (REF: BIL099100) were used to assess both direct and total bilirubin levels^35^. We used a commercial kit from SPECTRUM, Egypt, called ALB210002, which uses a modified bromocresol green colorimetric approach, to test albumin levels^36^. A kit from BIOMED Diagnostics in Oberschleißheim, Germany (Ref: ALP101050), was used to determine alkaline phosphatase (ALP) levels. The absorbance at 405 nm was determined to track the ALP activity throughout time.

#### Determination of kidney functions

A creatinine measurement kit from BIOMED Diagnostics in Oberschleißheim, Germany (Ref: CRE106240) was used^33^. Spectra (Egypt) (REF: 323,001)^37^ supplied the uric acid kits. The standard alkaline picric acid technique was used to test creatinine. This process involves reacting to picric acid with creatinine in an acidic solution to produce a complex that is yellow orange in color. At 492 nm, this compound was found to have a wavelength. Creatinine concentration is a good indicator of color intensity. It was SPECTRUM, Egypt that supplied the uric acid determination kit (with the product number 323001). The presence of 4-aminoantipyrine allows the colorimetric measurement of the uric acid content via the action of the enzymes uricase and peroxidase. It was at a wavelength of 546 nm when the quinone’s red pigment was detected.

#### Determination of lipid profile

From company FAR Diagnostics (Via Fermi, Italy), we received cholesterol (CHO), triglycerides (TG), high- and low-density lipoproteins (HDL-C and LDL-C), and total lipids kits.

#### Determination of oxidative stress markers in hepatic and renal tissues

Oxis Research TM, USA, supplied the MDA (malondialdehyde) kit. Eagle Diagnostics of Texas, USA, supplied the reduced glutathione (GSH) and superoxide dismutase (SOD) synthesis kits. The liver and kidneys were homogenized in 0.1 M phosphate buffer (pH 7.4),) and then minced with 10% water by weight. The homogenates were centrifuged at 12,000 × g for 30 min. at 4 degrees, the solution that was obtained was then analyzed. Sporadic, glutathione, and malondialdehyde concentrations were determined using the gathered homogenates. The MDA test kit was used to conduct a thiobarbituric acid reaction, which is a measure of lipid peroxidation. A crimson complex was produced, and its Nano Drope spectrophotometer reading came out at 532 nm. As stated in the reference^38^, the minimum concentration needed to detect MDA was 0.1 μM. Superoxide dismutase (SOD) was measured using an enzyme that converts superoxide anion to oxygen and hydrogen peroxide; glutathione (GSH) was measured by colorimetric analysis at 412 nm with a 0.1 mM detection limit^39^.

#### Pharmaceutical properties of endophytic *Fusarium equiseti*

We used the server Swiss ADME (http://www.swissadme.ch/index.php) to conduct ADMET analysis on the phytochemicals included in the MEE extracts. We examined and analyzed compounds’ physicochemical characteristics, including lipophilicity, hydrophilicity, pharmacokinetics behavior, drug likeness, and profiles of medicinal chemistry, using this service. Additionally, online servers known as Protox-II (https://tox-new.charite.de/protox_II/) ^40^ and StopTox (https://stoptox.mml.unc.edu/) ^41^ were used to determine the toxicity of the phytochemicals that were discovered. For the discovered ligands, Protox-II predicted the LD50 values (mg/kg) and toxicity categories, in contrast to StopTox, which predicted the risk of hERG and found cardiac toxicity.

#### Histopathological analysis

Hepatic and renal tissues were initially immersed in Bouin’s solution and subsequently transferred to 70% ethanol for preservation. Tissue dehydration was achieved using ascending ethanol concentrations, and tissues were then cleared in xylene. Samples were embedded in paraffin wax and sliced at a thickness of 4 µm using a rotary microtome. The paraffin sections were dewaxed in xylene for 20 min, then rehydrated through descending ethanol concentrations. Hematoxylin and eosin (H&E) staining was conducted using a tissue processor (ASP300s, Leica Biosystems, IL, USA). Histological evaluation was carried out under an Olympus BX 52 microscope and captured using a Canon DP21 digital camera mounted on the microscope ^42^ .

#### Statistical analysis

Statistical analysis was done using GraphPad Prism 8.0.1 (San Diego, CA, USA). Results (n = 5) were expressed as the mean ± SE Group comparisons were conducted via one-way analysis of variance (ANOVA) followed by Tukey’s post hoc test, with significance set at p < 0.05. symbols (^*^) indicated significance versus control, while symbols (^**#**^) denoted significance versus the CdCl_2_-treated group.

## Results

### Isolation of endophytic fungi from wild *Hyoscyamus muticus*

Eight isolates of five genus; *Aspergillus spp., Alternaria spp., Penicillium sp., Fusarium sp.* and *Cladosporium sp.* were isolated from *H. muticus* and identified according to their morphological characters*. Fusarium species* were selected for preparation of fungal extract. The morphological characters were confirmed by identification of AUMC. *Fusarium species* rapid in their growth, pale brown mycelium, pale pink reverse colony, one septum microconidium and 3–4 septum macroconidium (Fig. 1b).

### Genotypic identification

Through morphological inspection and molecular biological study, the fungal isolate *Fusarium equiseti* AUMC 15585 was determined to be *Fusarium equiseti* (*F. equiseti*). Using conventional fungal DNA isolation techniques, genomic DNA was isolated from culture filtrates in order to conduct genetic analysis at the Molecular Biology Research Unit, Assiut University. The ITS region of ribosomal DNA (rDNA) was amplified and sequenced with the aim of reliably identifying fungal species, taking into account the sequence diversity. We used the Basic Local Alignment Search Tool (BLAST) to compare the ITS sequence to sequences included in the NCBI GenBank database. Alignment results show that *F. equiseti* AUMC 15585 is a member of the *F. equiseti* species, with 99.61%-100% sequence identity and 99%-100% coverage compared to other reference strains of the same species. After validation, the resulting sequence was formally submitted to GenBank accession no. OP694170 and is available as a reference for comparative genomic studies and taxonomy. A phylogenetic tree was constructed using ITS sequences of closely related *F. equiseti* strains available in GenBank to further clarify its evolutionary position. Isolate fungal strain *F. equiseti* AUMC 15585 clustered into a well-supported *F. equiseti* lineage, confirming its genetic relatedness to previously identified strains. The remarkable similarity of the fungal strains identified in this study and their distinct positions in the phylogenetic tree justify this classification.

**Fig. 2:**
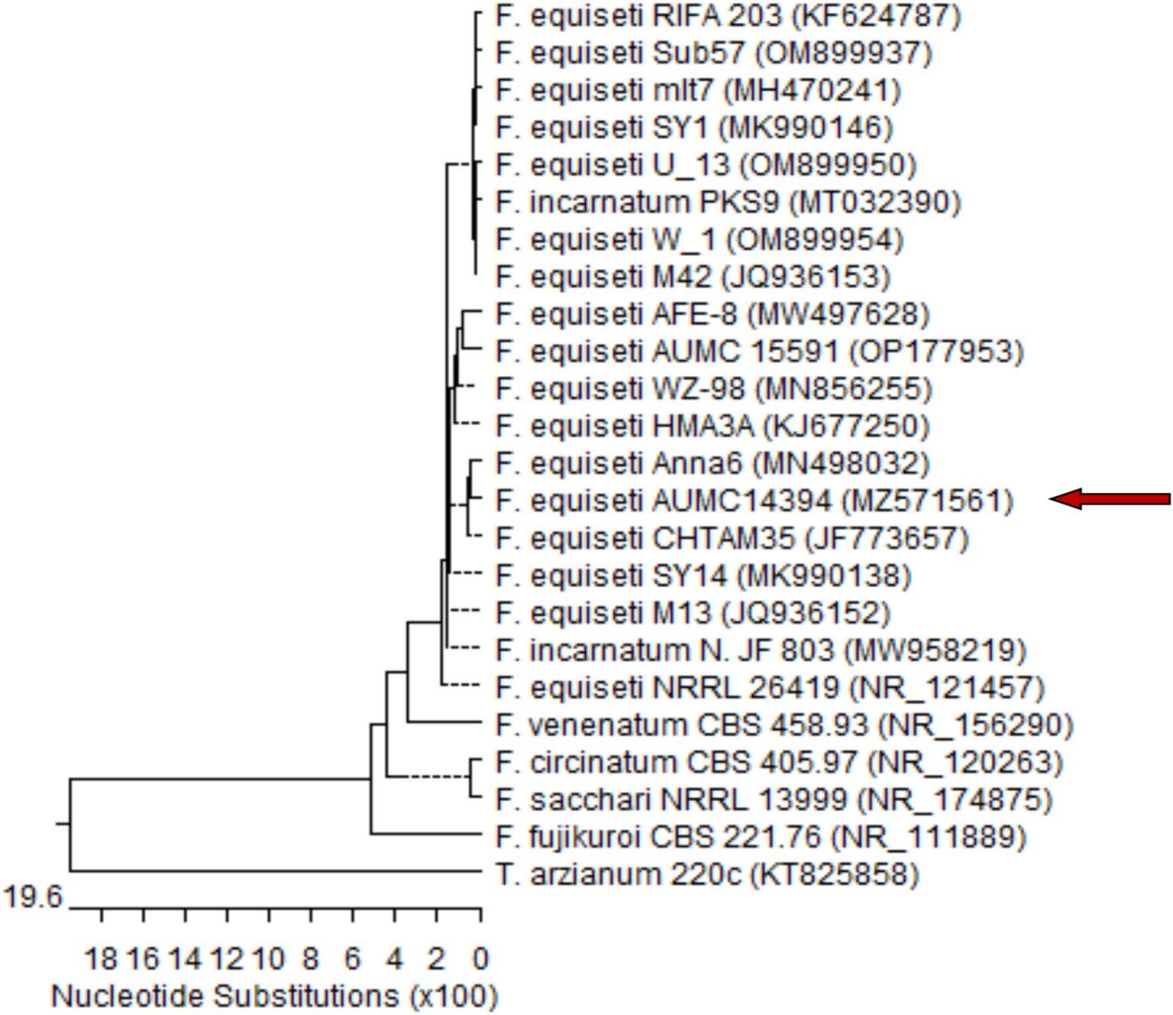
Phylogenetic tree based on ITS sequencing of rDNA of the fungal strain *Fusarium equiseti* AUMC 15585 with GenBank accession no. OP694170 (arrowed), aligned with closely related sequences of fungal strains accessed from the GenBank. F = *Fusarium*, A = *Aspergillus* (outgroup strain). *Fusarium equiseti* showed 99.62%—100% identity and 99%- 100% coverage with several strains of *F. equiseti* including the type of strain *F. equiseti* NRRL 26419 (NR_121457).

This study demonstrates the utility of molecular identification, sequence alignment, and ITS-based phylogenetic analysis in the classification of fungal species. The successful sequence deposition in GenBank will enrich the gene database and facilitate future comparative and functional studies on *F. equiseti* (Fig. 2), which is known for its ecological importance and production of secondary metabolites.

### GC–MS data analysis

**Fig. 3:**
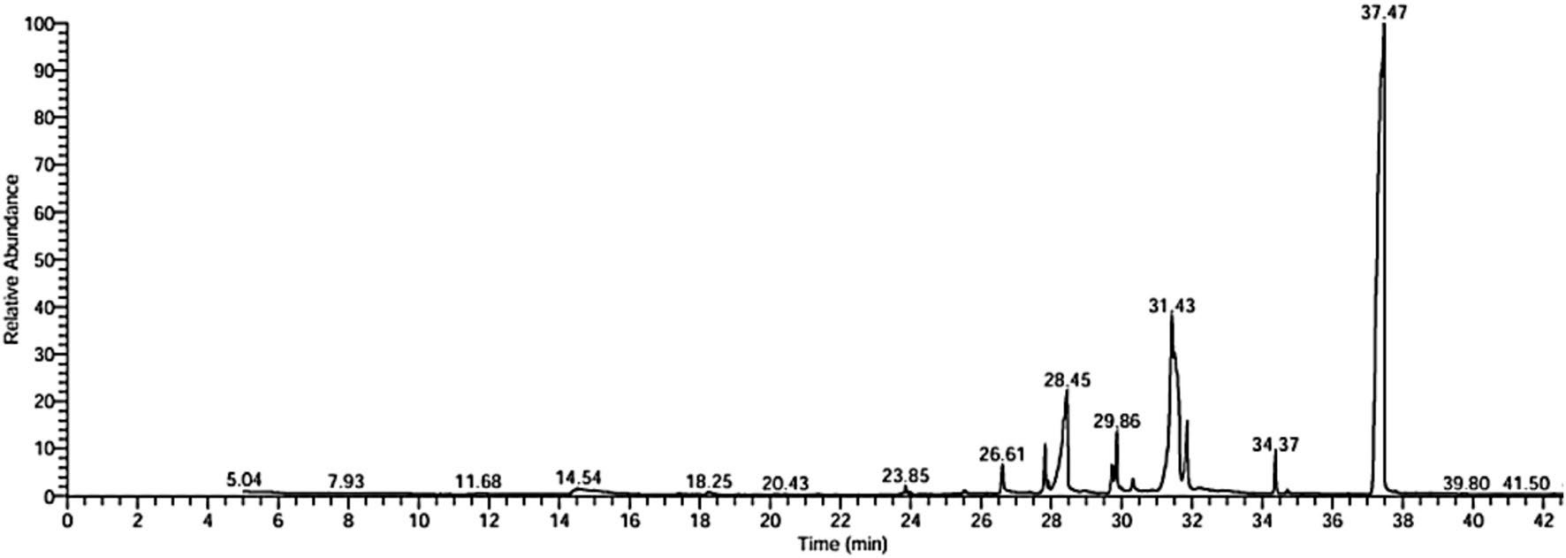
GC–MS chromatogram of the FE extract.

**Fig. 4:**
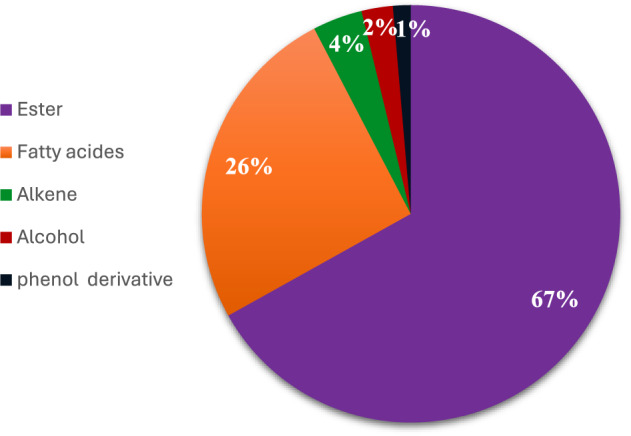
Classification of the phytochemical components of FE extract .

The metabolic components of FE extract were identified using GC–MS. As a result, 17 types of compounds were identified. The phytochemicals of FE were classified into esters, fatty acids, alkenes, alcohols, and phenol derivatives (Table 1 and Figs. 3 and 4). As a result, esters (67%) were the most abundant, followed by fatty acids (26%), alkenes (4%), alcohol (2%), and phenol derivatives (1%). The Bis (2-ethylhexyl) phthalate is the higher compound in RT (37.46 min) and in the area (63.51%), followed by 9,12-Octadecadienoic acid (Z,Z) (31.51 RT, 10,21%) and Oleic Acid (9-Octadecenoic acid, Z) (31.43 RT, 8.13%). These metabolites mainly play important role and are responsible for the antimicrobial activity of fungal extract.

**Table 1:** Identified compounds from GC–MS analysis of FE extract.

NO	RT (min)	Compound name	Molecular formula	Molecular weight (g/mol)	Area (%)	Biological effects	References
Ester 1 23.85		4-Trifluoroacetoxypentadecane	C_17_H_31_F_3_O_2_	324	0.35	No activity reported	-
2	26.61	Pentadecanoic acid, 14-methyl-, methyl ester	C_17_H_34_O_2_	270	1.23	Antimicrobial and antifungal properties	^43^
3	26.61	Hexadecanoic acid, methyl ester	C_17_H_34_O_2_	270	1.23	Antioxidant, anti-inflammatory, and antimicrobial activities	^16,44–46^
4	29.73	9,12-Octadecadienoic acid, methyl ester	C_19_H_34_O_2_	294	1.38	Antioxidant, anti-cancer, and anti-inflammatory properties	^47^
5	29.86	9-Octadecenoic acid (Z)-, methyl ester	C_19_H_36_O_2_	296	2.53	Antioxidant, antimicrobial, anticarcinogenic, antihypertensive agent	^48,49^
6	30.32	Octadecanoic acid, methyl ester	C_19_H_38_O_2_	298	0.49	Anti-tumor, cytotoxic and antimicrobial, antioxidant, and anti-inflammatory effects	^50^
7	37.46	Bis(2-ethylhexyl) phthalate	C_24_H_38_O_4_	390	63.51	Antimicrobial, cytotoxic, caspase 3 stimulant, transcription factor NF kappa B stimulant, transcription factor stimulant, and antineoplastic effects	^51,52^
alkene 8 23.85		10-Heneicosene (c,t)	C_21_H_42_	294	0.35	Antibacterial activity	^53^
9	27.82	1-Docosene	C_22_H_44_	308	1.86	Antibacterial and antioxidant activities	^54^
10	27.82	Heptacos-1-ene	C_27_H_54_	378	1.86	Antimicrobial and antioxidant activity (as part of complex natural extracts in GC‑MS profiling)	^55^
Fatty acids 11 28.45		n-Hexadecanoic acid (palmitic acid)	C_16_H_32_O_2_	256	5.60	Anti-inflammatory, antioxidant, antibacterial, anticancer, hepatoprotective, anti-coronary, and hypocholesterolemic effects	^46,56,57^
12	31.43	Oleic Acid (9-Octadecenoic acid, Z)	C_18_H_34_O_2_	282	8.13	Antibacterial and antioxidant effects	^16^
13	31.51	9,12-Octadecadienoic acid (Z,Z)	C_18_H_32_O_2_	280	10.21	Anti-inflammatory, hepatoprotective, hypocholesterolemic, cancer-preventive, anti-coronary, and hypocholesterolemic effects	^46^
14	31.87	Octadecanoic acid (Stearic acid)	C_18_H_36_O_2_	284	2.98	Antibacterial, anti-inflammatory, antioxidant, and anticancer activities	^44,45,57,58^
alcohol 15 23.85		1-Hexadecanol, 2-methyl-	C_17_H_36_O	256	0.35	Identified in GC‑MS profiles of essential oils and extracts that exhibit antimicrobial activity	^59^
16	23.85	1-Eicosanol	C_20_H_42_O	298	2.21	Antimalarial, antioxidant, antifungal	^60^
phenol derivative 17 34.37		[1,1’-Biphenyl]-2,3’-diol derivative	C_28_H_42_O_2_	410	1.44	Antibiotic, antifungal, antioxidant, anti-cancer, anti- inflammatory, and anti-aging agent	^61,62^

### Biological evaluation of the *Fusarium equiseti* secondary metabolites ethyl acetate extract (FE)

#### Antioxidant activity

**Fig. 5:**
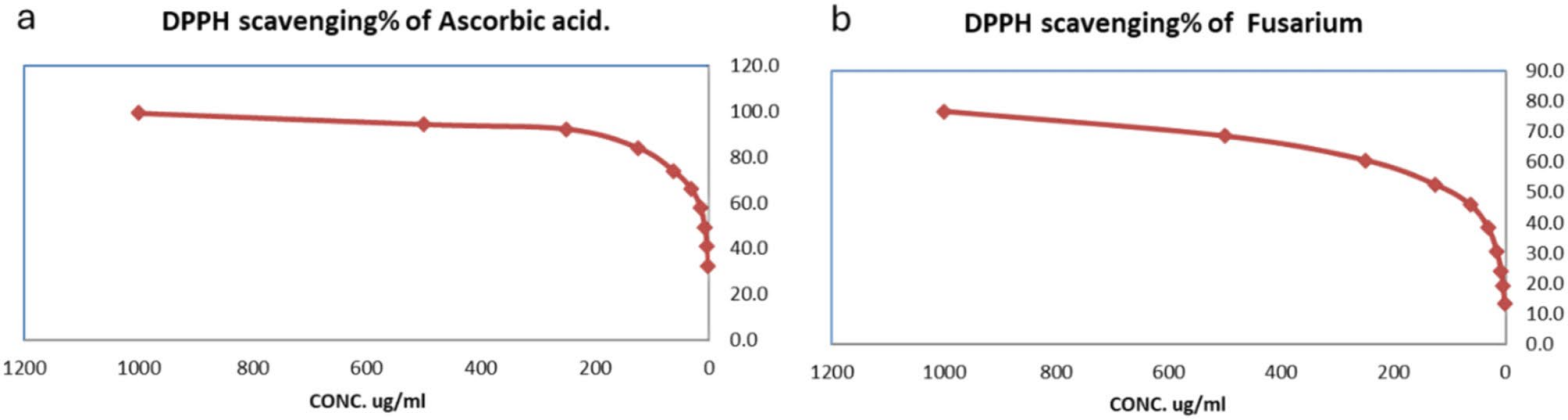
DPPH Radical Scavenging Activity of Ascorbic Acid (**a**) and FE extract (**b**).

The antioxidant activity of FE extract increased with increasing concentrations in the DPPH radical scavenging test, demonstrating dose-dependence. The extract demonstrated its capacity to neutralize free radicals at a concentration of 1000 µg/mL, as shown in Table 3, with a scavenging activity of 71.3 ± 0.003%. Despite this, it never had a greater impact than ascorbic acid, which demonstrated 99.1 ± 0.004% scavenging activity at the same dosage, proving that it is an effective antioxidant (Table 2, Fig. 5a&b). The IC50 results highlighted the difference in scavenging capacity between the two samples:  FE extract required 145.91µg/mL to inhibit radicals by 50%, while ascorbic acid showed significantly stronger activity with an IC50 of 8.041µg/mL. This stark contrast suggests that FE extract does contain bioactive metabolites that scavenge reactive species, but with lower potency than standard antioxidants such as ascorbic acid. At intermediate concentrations, the extract maintained moderate activity, showing an absorbance of 46.8 ± 0.002% at 125 µg/mL, which then gradually decreased to 9.2 ± 0.003% at 1.95 µg/mL. Meanwhile, ascorbic acid consistently outperformed the extract at all concentrations tested, showing a higher electron donating capacity.

**Table 2:** DPPH radical scavenging activity of FE extract and ascorbic acid.

Concentration (µg/mL)	FE extract (% Scavenging ± SD)	Ascorbic acid (% Scavenging ± SD)
1000	71.3 ± 0.003	99.1 ± 0.004
500	63.7 ± 0.001	94.2 ± 0.006
250	55.7 ± 0.003	92.2 ± 0.004
125	46.8 ± 0.002	84.0 ± 0.014
62.5	39.1 ± 0.004	73.9 ± 0.006
31.25	32.3 ± 0.005	65.9 ± 0.007
15.625	27.2 ± 0.008	57.9 ± 0.005
7.8125	21.0 ± 0.005	49.1 ± 0.008
3.9	15.4 ± 0.007	41.1 ± 0.006
1.95	9.2 ± 0.003	32.5 ± 0.021
IC₅₀ (µg/mL)	145.91	8.041

#### Antimicrobial activity

The antimicrobial activity of FE extract was measured using the agar well diffusion technique against the selected studied organisms. The 6.0 mm well diameter was filled with 100 µL of samples dissolved in 0.9% DMSO, and then three replicates of fungal extracts were performed at a concentration of 10 mg/mL. For comparison of inhibition zones, the control antibiotic gentamicin (4 µg/mL) was used. Gram-positive (*Bacillus subtilis*, *Staphylococcus aureus*), Gram-negative (*Escherichia coli*, *Pseudomonas aeruginosa*), and pathogenic yeast (*Candida albicans*), and fungi (*Aspergillus fumigatus*) were tested. The antimicrobial effect of the positive control, gentamicin (4 µg/mL), against the studied organisms varied but was lower than the effect of the fungal extract. The absence of antimicrobial activity of DMSO (negative control) against the tested pathogens proved that any inhibition observed was caused only by the fungal extract.

Filamentous fungus *A. fumigatus * showed no sensitivity to FE extract, indicating that it was unaffected by the extract’s bioactive metabolites or that the agar well diffusion technique was not suitable for filamentous fungi. The positive control (gentamicin) recorded inhibitory effects against the studied organisms as follows: 22, 15, 17, and 16 mm for *B. subtilis* (ATCC 6633), *S. aureus* (ATCC 6538), *E. coli* (ATCC 8739), and *P. aeruginosa* (ATCC 90274), respectively, and 23 and 20 mm for *C. albicans* (ATCC 10221) and *A. fumigatus *, respectively.

In contrast, the inhibition zones caused by FE extract against the same organisms under the same controlled conditions confirmed that the FE extract exhibited antibacterial activity (34, 30, 46, and 40 mm, respectively) and an inhibitory effect against *C. albicans* (38 mm) compared with the positive control (Table 3). *A. fumigatus*, on the other hand, showed no signs of activity, indicating that it was either resistant to or unaffected by the extract’s bioactive metabolites.

**Table 3:** Antimicrobial activity of FE extract.

Pathogenic microorganism	Inhibition zone (mm)	Control (Gentamycin, 4 µg/mL for bacteria or ketoconazole, 100mg/mL for fungi) (mm)
*Bacillus subtilis* (ATCC 6633)	34	22
*Staphylococcus aureus* (ATCC 6538)	30	15
*Escherichia coli* (ATCC 8739)	46	17
*Pseudomonas aeruginosa* (ATCC 90,274)	40	16
*Candida albicans* (ATCC 10,221)	38	23
*Aspergillus fumigatus *	NA	20

#### Anti-inflammatory activity

The evaluation of anti-inflammatory properties *in vitro *frequently employs the human red blood cell (HRBC) membrane stabilization assay, commonly measured by the inhibition of induced hemolysis (lysis). The data presented in Table 4 evaluates the concentration -dependent activity of FE extract in inhibiting hemolysis, quantified by measuring the absorbance of hemoglobin released into the supernatant under induced lytic conditions. The results demonstrate a clear, positive correlation between the concentration of the fungal extract and its protective effect on the red blood cell membranes.

**Table 4:** Hemolysis inhibition activity of FE extract.

Sample (Conc. µg/mL)	Absorbance (isotonic solution)	Hemolysis Inhibition (% ± SD)
Control	-	0.0 ± 0.049
1000	0.098	55.9 ± 0.009
800	0.069	47.0 ± 0.008
600	0.052	35.0 ± 0.007
400	0.034	22.2 ± 0.011
200	0.021	12.0 ± 0.011
100	0.021	3.1 ± 0.031

The extract of FE inhibited hemolysis in a concentration-dependent manner, suggesting its involvement in stabilizing the erythrocyte membrane. The control group showed a 0% inhibition rate, confirming the protective effect of the extract (Table 4). As the extract concentration increases, the percentage of hemolysis inhibition rises substantially. At the lowest tested concentration 100 µg/mL, the inhibition is marginal (3.1 ± 0.031%). However, this protective effect accelerates, reaching 22.2 ± 0.011% at 400 µg/mL and achieving a maximum inhibition of 55.9 ± 0.009%, at the highest tested concentration of 1000 µg/mL. The consistent, stepwise increase in protection across the concentration gradient (100 µg/mL to 1000 µg/mL) confirms that the anti-hemolytic principles are present in biologically significant amounts and exert a predictable pharmacological effect. This membrane-stabilizing effect suggests that bioactive metabolites in the extract neutralize osmotic stress and prevent erythrocyte lysis.

###  *In vitro* cytotoxicity and Selectivity Index (SI) of FE extract 

**Fig. 6:**
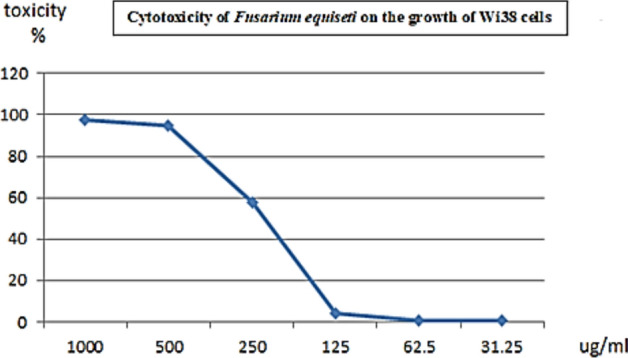
Cytotoxicity of FE extract on the growth of Wi38 cells were examined by MTT assay. Dose response curves constructed in the range 1000–31.25 µg/mL after 24 h.

**Fig. 7:**
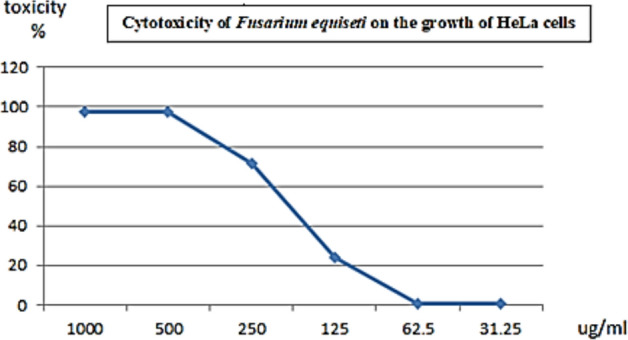
Cytotoxicity of FE extract on the growth of HeLa cells were examined by MTT assay. Dose response curves constructed in the range 1000–31.25 µg/mL after 24 h.

**Fig. 8:**
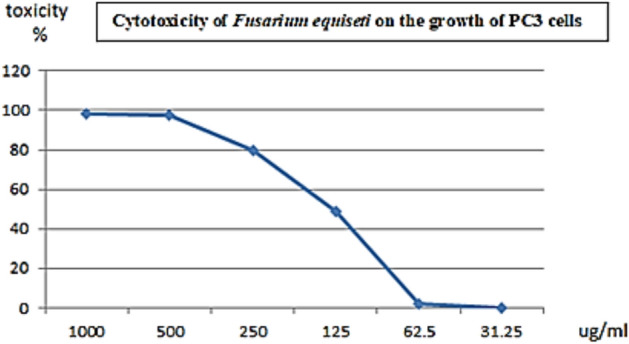
Cytotoxicity of FE extract on the growth of PC3 cells were examined by MTT assay. Dose response curves constructed in the range 1000–31.25 µg/mL after 24 h.

 FE extract showed dose-dependent cytotoxicity towards WI38, HeLa, and PC3 cells (Table 5, Figs. 6, 7 & 8), using MTT assay in the absence and presence of the crude extract of multiple concentrations. There was a dose-dependent response when six different concentrations (31.25, 62.5, 125, 250, 500 and 1000 µg/ml) were tested for each sample. The results of the cytotoxic activity of fungal crude extracts are shown in Table 5. The present study revealed that the IC50 (concentration of the extract that decreases the number of viable cells by 50%) value of FE extract was 194.15 µg/ml, 162.88 µg/ml, and 237.26 µg/ml for HeLa, PC3, and WI38 respectively. The IC50 values for HeLa and PC3 fall within the moderate cytotoxicity category for crude natural product extracts, which generally range from around 100–200 µg/ml depending on the source. This moderate cytotoxicity confirms the potential of the extract as anticancer agent and is consistent with fusarium species being prolific producers of diverse bioactive metabolites with protective potential. The extract exhibits greater potency against the PC3 human prostate cancer line compared to HeLa cervical cancer cells, suggesting that its active compounds may specifically interfere with molecular pathways crucial to PC3 proliferation or survival. This behavior reflects how certain fusarium metabolites have been reported to show selective anticancer activities depending on cancer cell type. Regarding toxicity to normal cells, the IC50 for the non-malignant WI38 lung fibroblast line is 237.26 µg/m, indicating less sensitivity and thus favorable safety profile. This selectivity is desirable as it implies the extract can target cancer cells over normal cells, reducing potential side effects.

**Table 5:** Cytotoxic activity and selectivity index of FE extract.

Cell line	*Wi38*		*HeLa*		*Pc3*	
*Concentration* *(µg/mL)*	Viability (%)	Toxicity (%)	Viability (%)	Toxicity (%)	Viability (%)	Toxicity (%)
*Control*	**100.00**	**0.00**	**100.00**	**0.00**	**100.00**	**0.00**
*1000*	2.62	97.38	2.60	97.39	2.11	97.89
*500*	4.88	95.12	2.71	97.29	2.61	97.39
*250*	42.65	57.35	28.92	71.08	20.24	79.76
*125*	95.67	4.33	76.24	23.76 4	51.55	48.45
*62.5*	99.24	0.76	99.36	0.64	98.29	1.71
*31.25*	99.04	0.96	99.20	0.8	99.87	0.13
*IC₅₀ (µg/mL)*	237.26		194.15		162.88	
Selectivity Index (SI*)			1.22		1.46

The selectivity index values calculated as the ratio of IC50 in normal cells to IC50 in cancerous cells are approximately 1.22 for HeLa and 1.46 for PC3. SI values greater than one signify a protective window favoring cancer cell eradication while sparing normal cells, supporting the extract’s potential as anti-cancer agent with selective toxicity. Images captured under a microscope showed that the cytotoxic impact of FE extract on WI38, HeLa, and PC3 cells was dose dependent (S1, 2 &3). The cells in group A, which did not receive any treatment, maintained their elongated shape and strong adhesion. Nevertheless, the cells progressively detached, shrunk, and became less dense as the concentration of the extract (B) rose; the concentration of 1000 µg/mL caused the most severe damage. Below 250 µg/mL, some viable cells remained but some cytotoxicity was observed. These results suggest that the extract may disrupt cellular integrity and induce apoptotic pathways or membrane instability, and its bioactive compounds and mechanisms require further investigation.

### The impact of FE extract (100mg/kg) on body and relative organ weight in rats treated with CdCl_2_

To assess the physiological impact of CdCl_2_ (3 mg/kg) and FE (100 mg/kg) over 14-day treatment period, the data presented in Table 6 were examined. Throughout the study, no statistically significant differences (P < 0.05) were observed in body weight or relative organ weights between any treatment group and the control group.

**Table 6:** Effect of FE extract (100 mg/kg) on body and relative organs weight in rats treated with CdCl_2_.

Parameters	Control	CdCl_2_	FE	FE + CdCl_2_
Initial weight (gm)	173.5 ± 4.9	176.5 ± 6.6	168.0 ± 7.2	165.3 ± 3.1
Final weight (gm)	211.0 ± 2.3	219.0 ± 5.2	188.0 ± 9.2	183.0 ± 3.5
Liver (%)	2.8 ± 0.03	2.7 ± 0.2	2.8 ± 0.1	2.7 ± 0.04
R. kidney (%)	0.3 ± 0.03	0.4 ± 0.01	0.4 ± 0.01	0.3 ± 0.01
L. kidney (%)	0.6 ± 0.1	0.5 ± 0.08	0.4 ± 0.002	0.4 ± 0.07
R. Testis (%)	0.9 ± 0.03	0.8 ± 0.09	1.1 ± 0.1	0.7 ± 0.02
L. Testis (%)	1.0 ± 0.06	0.1 ± 0.1	1.1 ± 0.07	0.7 ± 0.02
Heart (%)	0.4 ± 0.04	0.4 ± 0.0	0.4 ± 0.0	0.3 ± 0.01
Spleen (%)	0.2 ± 0.006	0.2 ± 0.03	0.2 ± 0.0	0.18 ± 0.01

Data were expressed as mean ± SE (n = 5), analyzed by one-way ANOVA then by Tukey’s test. Values with different symbols (* versus control group, versus CdCl_2_-treated group) differ significantly at P < 0.05. G I: Control group; G II: CdCl_2_-treated group; G III: secondary metabolites extract of *Fusarium* group; G IV: secondary metabolites extract of *Fusarium* / CdCl_2_ group.

### The impact of FE extract (100mg/kg) on hematological parameters in rats treated with CdCl_2_

After 14 days of being administered CdCl_2_ (3 mg/kg) and FE (100 mg/kg) to rats, the findings of hematological profile are shown in Table 7. Not all hematological parameters were altered by 14 days of FE treatment. At the same time, as compared to the control group, the counts of white blood cells and lymphocytes were considerably elevated (P < 0.05) after intraperitoneal injection of CdCl_2_. In contrast to the CdCl_2_ group, the white blood cell count was considerably reduced (P < 0.05) in the FE + CdCl_2_ treated group.

**Table 7:** Effect of FE extract (100 mg/kg) on hematological parameters in rats treated with CdCl_2_.

Parameters	Control	CdCl_2_	FE	FE + CdCl_2_
RBCs (× 10^6^ /mm^3^)	7.0 ± 0.2	6.9 ± 0.4	7.4 ± 0.3	6.9 ± 0.07
HB g/dL	13.5 ± 0.4	13.3 ± 0.7	13.9 ± 0.4	13.3 ± 0.2
HCT %	41.3 ± 1.4	40.6 ± 2.6	43.4 ± 1.9	40.8 ± 0.4
MCV fL	58.4 ± 0.6	58.5 ± 0.8	58.4 ± 0.1	59.1 ± 0.8
MCH pg	19.1 ± 0.1	19.1 ± 0.03	18.7 ± 0.2	19.3 ± 0.4
MCHC g/dL	32.7 ± 0.5	32.6 ± 0.3	33.5 ± 0.1	32.6 ± 0.2
WBCs (× 10^3^/mm^3^)	11.52 ± 0.57	17.02 ± 1.16**	12.00 ± 0.61	11.14 ± 0.78^**##**^
LYM (%)	5.6 ± 0.8	8.7 ± 0.5*	5.2 ± 0.03	6.6 ± 0.6
PLT (× 10^3^/mm^3^)	1023 ± 2.6	943.3 ± 79.2	817.3 ± 2.3	902.3 ± 90.1

Data were expressed as mean ± SE (n = 5), analyzed by one-way ANOVA then by Tukey’s test. Values with different symbols (* versus control group, ^#^ versus CdCl2-treated group) differ significantly at P < 0.05. G I: Control group; G II: CdCl_2_-treated group; G III: secondary metabolites extract of *Fusarium* group; G IV: secondary metabolites extract of *Fusarium* / CdCl_2_ group.

### Serum fasting glucose (FBG) and liver markers in different experimental groups

**Fig. 9:**
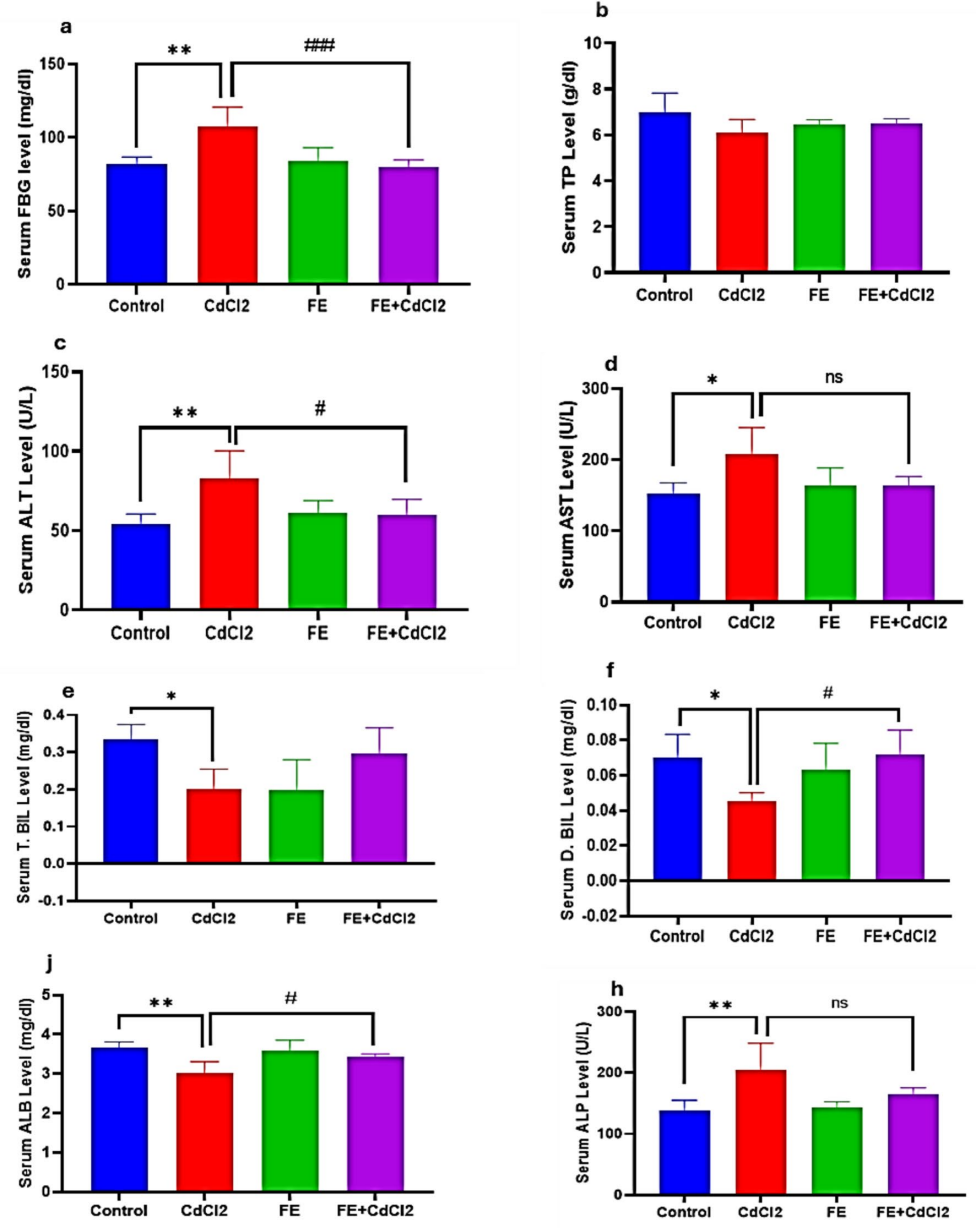
Effect of FE extract (100 mg/kg) on serum glucose and liver markers in rats treated with CdCl_2_.

Final experimental results for fasting glucose and liver indicators in the control and treated groups are shown in Fig. (9). Compared to the control group, the CdCl_2_ treated group showed considerably higher levels of fasting glucose (Fig. 9a), ALT (Fig. 9c), AST (Fig. 9d), and ALP (Fig. 9h) (P < 0.05). Conversely, levels of T. BIL (Fig. 9e), D. BIL (Fig. 9f), and ALB (Fig. 9g) were markedly reduced. Notably co-administration of FE extract with CdCl_2_ further enhanced these alterations. On contrast the group treated with FE only showed no significant changes in any of the measured parameters relative to the control group (P < 0.05).

### Effect of FE extract (100mg/kg) on serum kidney function in rats treated with CdCl_2_.

**Fig. 10:**
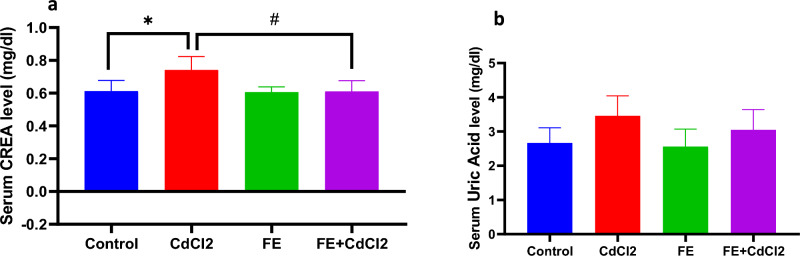
Effect of FE extract (100 mg/kg) on Serum Kidney Functions in Rats Treated with CdCl_2_.

Serum creatinine levels in rats that were given 3 mg/kg of CdCl_2_ intraperitoneally rose considerably (P < 0.05). Figure 10a shows that cadmium-induced renal damage was much ameliorated and serum creatinine levels dropped when FE extract was also given. Uric acid levels, nevertheless, were unchanged across all treatment groups (Fig. 10b).

### Effect of FE extract (100mg/kg) on Serum Lipid Profile in Rats Treated with CdCl_2_

As shown in Table 8, various treatments exerted adverse effects on the lipid profile. TG levels decreased in the FE-only group as compared to the control group. In contrast, administration of CdCl_2_ significantly elevated levels of total lipids, triglycerides, CH, and LDL-C while reducing levels of HDL-L. In contrast notably, co-administration of FE extract with CdCl_2_ effectively stabilized total lipids, triglycerides, CH, and LDL-C levels, resulting in marked improvement in all lipid profile parameters.

**Table 8:** Effect of FE extract (100 mg/kg) on Lipid Profile in rats treated with CdCl_2_.

Parameters	Control	CdCl_2_	FE	FE + CdCl_2_
Total Lipids (mg/dl)	248.4 ± 16.10	314.4 ± 15.58*	211.8 ± 2.31	259.6 ± 12.25^**#**^
TG (mg/dl)	96.40 ± 1.66	118.6 ± 3.37***	63.60 ± 3.12***	97.40 ± 2.71^**###**^
CH (mg/dl)	76.40 ± 1.57	95.00 ± 3.35***	70.80 ± 0.92	76.00 ± 3.83^**###**^
HDL-C (mg/dl)	33.40 ± 2.54	19.60 ± 1.12***	27.80 ± 2.08	22.20 ± 1.36
LDL-C (mg/dl)	43.20 ± 3.66	61.00 ± 2.92**	40.40 ± 3.03	49.80 ± 1.36

Data were expressed as mean ± SE (n = 5), analyzed by one-way ANOVA then by Tukey’s test. Values with different symbols (* versus control group, ^#^versus CdCl_2_-treated group) differ significantly at P < 0.05. G I: Control group; G II: CdCl_2_-treated group; G III: secondary metabolites extract of *Fusarium* group; G IV: secondary metabolites extract of *Fusarium* / CdCl_2_ group.

### Effect of FE extract (100mg/kg) on Oxidative Stress Markers in rats treated with CdCl_2_

The activity of antioxidant markers and malondialdehyde (MDA) concentrations across the different treatment groups are summarized in Table 9. MDA, a lipid peroxidation by-product of polyunsaturated fatty acids, was measured in liver and kidney tissues as the MDA-2-thiobarbituric acid (TBA) conjugate, alongside measurements GSH, and SOD. Administration of CdCl_2_ resulted in a significant reduction in GSH and SOD concentrations and a marked elevation in MDA in both tissues. In contrast, the FE only treated group exhibited antioxidant levels markers similar to those in the control group. On the other hand, in cadmium-intoxicated rats, administration of 100 mg/kg FE decreased MDA concentration and significantly increased GSH and SOD concentrations in both tissues (p < 0.0001 compared with the CdCl_2_-treated group).

**Table 9:** Effect of FE extract (100 mg/kg) on Oxidative Stress Markers in rats treated with CdCl_2_.

Parameters	Control	CdCl_2_	FE	FE + CdCl_2_
Renal MDA (nmol/mg)	7.1 ± 0.5	15.3 ± 0.7****	5.4 ± 0.03	9.1 ± 0.4^**####**^
Renal GSH (U/mg)	22.6 ± 1.5	12.6 ± 0.4**	24.1 ± 1.0	23.0 ± 1.9^**##**^
Renal SOD (U/mg)	23.5 ± 0.8	13.0 ± 0.6***	27.0 ± 2.3	20.0 ± 0.6^**#**^
Hepatic MDA (nmol/mg)	9.07 ± 0.47	24.28 ± 0.82****	10.24 ± 0.77	14.44 ± 0.86^**####**^
Hepatic GSH (U/mg)	30.68 ± 1.07	20.44 ± 0.83***	29.92 ± 1.69	28.68 ± 1.21^**##**^
Hepatic SOD (U/mg)	38.84 ± 2.85	17.04 ± 0.59****	40.32 ± 0.85	25.72 ± 1.35^**##**^

Data were expressed as mean ± SE (n = 5), analyzed by one-way ANOVA then by Tukey’s test. Values with different symbols (^*^ versus control group, ^#^ versus CdCl_2_-treated group) differ significantly at P < 0.05. G I: Control group; G II: CdCl_2_-treated group; G III: secondary metabolites extract of *Fusarium* group; G IV: secondary metabolites extract of *Fusarium* / CdCl_2_ group.

### Renal and Hepatic Histopathology in Male Rats After CdCl_2_ and/or FE extract Administration

**Fig. 11:**
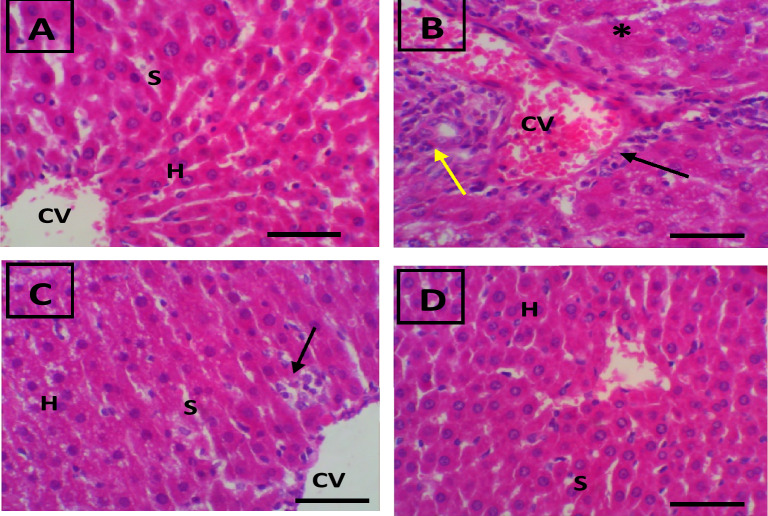
Photomicrographs of paraffin sections of the liver of (**A**) control, (**B**) CdCl_2_, (**C**) FE + CdCl_2_, (**D**) FE alone. Magnification: 400x; scale bar 50 µm.

Normal liver morphology was observed in the liver tissues of rats in the control group (A) and FE group (D). Normal hepatocytes had microvacuolated eosinophilic cytoplasm and round central vesicular nuclei with prominent nucleoli, and normal sinusoids were observed between hepatocytes. The sinusoids are lined with Kupffer cells. Hepatocytes are polygonal cells with a central nucleus arranged in radial rows separated by hepatic sinusoids lined with endothelial cells and Kupffer cells. In the CdCl_2_ group (B), structural and functional lesions in the liver were prominent. Hepatocellular lesions included complete disappearance of normal hepatic cord structure and marked hepatic necrosis. Hepatocytes had swollen vacuolated cytoplasm due to lipid accumulation, resulting in microvesicular or macrovesicular fatty liver. Some hepatocytes showed balloon-like degeneration with pale cytoplasm, suggesting severe cellular stress. Nuclear changes were prominent, with pyknosis, karyorrhexis, and karyoresis, and nuclear fragmentation or lysis in necrotic cells. Focal areas of hepatocellular necrosis and cell death were observed, especially in centrilobular regions with inflammatory cell infiltration. Sinusoidal endothelial cells were damaged, and hepatic microcirculation was impaired. Fibrosis was seen in portal and periportal areas. Additionally, capillary cholestasis was observed. In the FE + CdCl_2_ (C) group, the liver structure and histological findings were normal. Hepatocytes and sinusoids were almost normal, and thin inflammatory infiltration was observed (Fig. 11). Magnification: 400x; scale bar 50 µm.

**Fig. 12:**
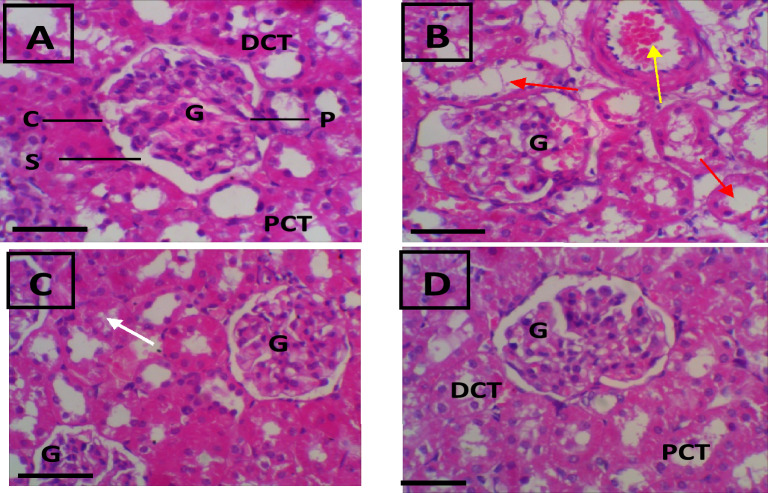
Photomicrographs of a paraffin section of the kidneys. (**A**) control, (**B**) CdCl_2_, (**C**) FE + CdCl_2_, (**D**) FE alone. Magnification: 400x; scale bar 50 µm.

The control group (A) and FE group (D) showed normal renal structure, with glomeruli surrounded by the glomerular capsule, normal capsular space and vascular pole, and normal proximal and distal convoluted tubules. The CdCl_2_ group (B) showed significant structural renal damage, mainly targeting the renal corpuscle and proximal tubule. The glomeruli showed signs of shrinkage, decreasing in size and cellularity. The basement membrane was thickened, capillaries collapsed, and filtration was impaired. The mesangial layer showed signs of expansion with increased matrix due to inflammation and injury. Interstitial edema was observed, with fluid accumulation in the interstitial spaces, and inflammatory cells were found in the renal interstitium around the glomeruli and tubules. The proximal and distal tubules showed focal areas of necrosis, with numerous obvious signs of nuclear changes, such as increased nuclear density, karyorrhexis, and karyorrhexis. Vascular damage was observed in the form of swelling of the endothelium of congested renal vessels, with capillary pooling and rupture causing focal hemorrhage. The FE + CdCl_2_ (C) group showed improvement in renal histology, similar to the control group, although a small number of swollen cells were still present (Fig. 12). Magnification: 400x; scale bar 50 µm .

### *In Silico* ADMET analysis of endophytic *Fusarium equiseti*

**Fig. 13:**
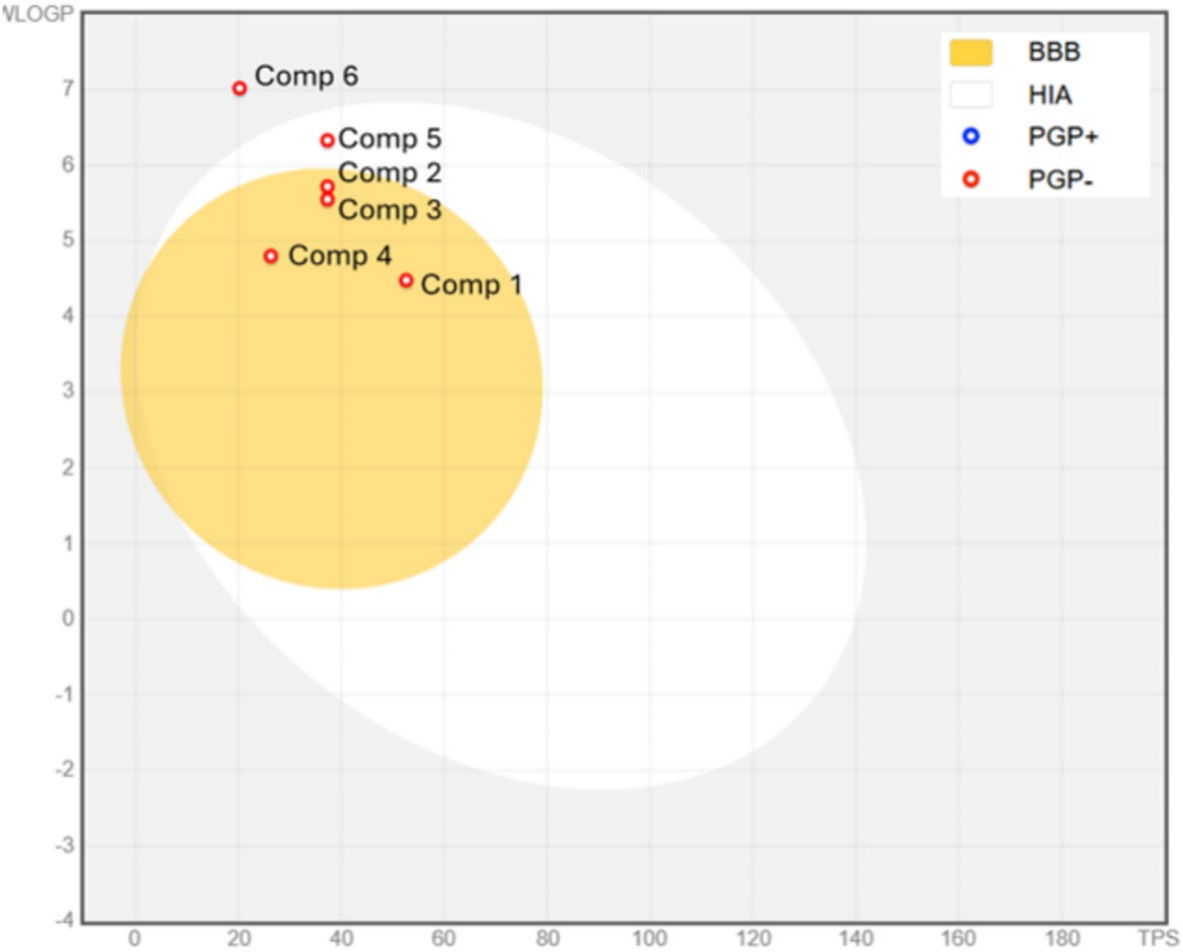
Boiled egg chart for the 6 compounds of the FE extract .

**Fig. 14:**
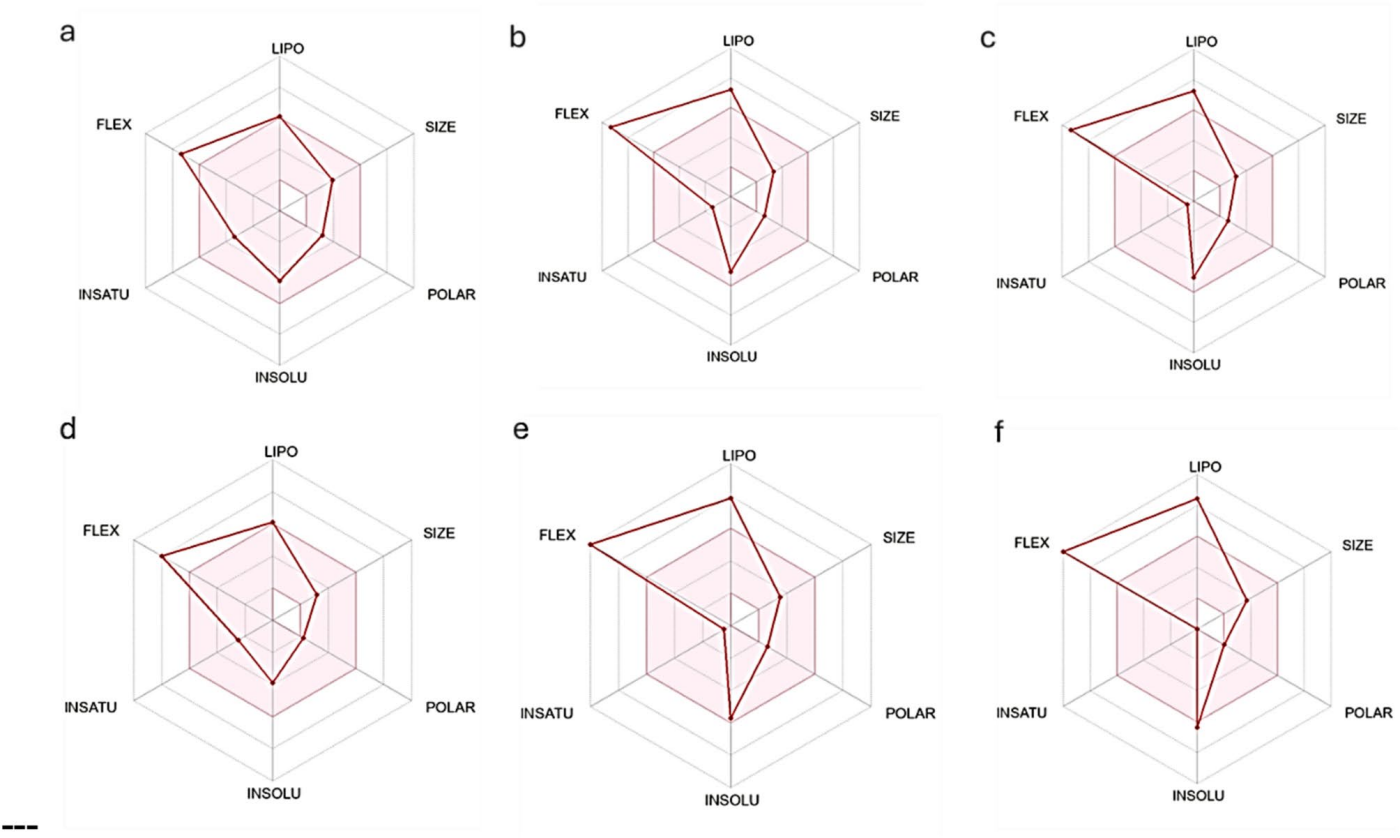
Bioavailability radar chart for the 6 compounds of the FE extract  .

The ADME profile of the six drugs shows important pharmacokinetic properties that may affect their efficacy (Supplementary Table S1). Compound 1 exhibits good oral bioavailability due to moderate solubility, high gastrointestinal absorption, and blood–brain barrier permeability; however, it inhibits CYP2C9 and CYP3A4, suggesting metabolic interactions. Compound 2 has improved permeability due to its high lipophilicity and rotatable bond but violates Lipinski’s rule due to its flexibility. As an inhibitor of CYP1A2 and CYP2C9, it suggests metabolic restrictions. Compound 3 has similar properties to compound 2, but has a lower molar refractive index, which affects intermolecular interactions. Compound 4 has a low TPSA and moderate solubility, which promotes passive diffusion while inhibiting hydrogen bonding. Compound 5, the most lipophilic, has good permeability but low solubility. Despite its high lipophilicity and turnover, compound 6 has limited blood–brain barrier (BBB) and gastrointestinal (GI) permeability, reducing its systemic availability. Not all drugs have P-gp efflux potential, which reduces drug tolerance; however, inhibition of metabolic enzymes is important. Bioavailability plots show the GI and BBB ​ permeability of six compounds. Yellow areas represent compounds that may cross the BBB, and white areas represent compounds with high intestinal absorption (HIA). Compounds 1–5 exhibited good gastrointestinal absorption and brain penetration, suggesting systemic and central activity. Compound 6, located outside the blood–brain barrier region, has limited brain penetration, limiting its neurological applications. All compounds labeled PGP- are not substrates of P-glycoprotein, reducing the risk of excretion and improving retention and bioavailability in the body (Fig. 13). The Bioavailability Radar Map evaluates key factors influencing the oral absorption of compounds 1, 2, 3, 4, 5, and 6. Lipophilicity is generally moderate to high, facilitating membrane permeability while limiting solubility. Molecular size varies, with some falling within and others exceeding the optimal range, which may impact absorption efficiency. The polarity mismatch is significant, and some chemicals are outside the optimal range, which may limit membrane permeability (Fig. 14). Solubility is an issue for some substances as reduced solubility can reduce adsorption capacity. Saturation levels affect the stability of the molecule and flexibility contributes to binding to the target. Some drugs have good absorption properties, while others may require modification or formulation to improve efficacy. Furthermore, cytotoxicity estimations have also been performed using *in silico* methods and are shown in Table 10.

**Table 10:** *In silico* toxicity analysis of phytochemicals tested.

No	Phytochemicals	Oral toxicity of phytochemicals (PROTOX II)		Acute Inhalation Toxicity	Acute Oral Toxicity	Acute Dermal Toxicity
		Predicted LD50 (mg/kg)	Predicted toxicity class			
1	Bis(2-ethylhexyl) phthalate	1190	4	Non-Toxic (94%)	Non-Toxic (99%)	Non-Toxic (98%)
2	9,12-Octadecadienoic acid (Z,Z)	1190	4	Non-Toxic (60.0%)	Non-Toxic (98.0%)	Non-Toxic (99.0%)
3	Oleic Acid (9-Octadecenoic acid, Z)	1190	6	Non-Toxic (60.0%)	Non-Toxic (98.0%)	Non-Toxic (99.0%)
4	n-Hexadecanoic acid	1190	4	Non-Toxic (56.0%)	Non-Toxic (100%)	Non-Toxic (99.0%)

## Discussion

In the North Sinai ecosystems, approximately 100 to 120 medicinal plant-endophyte associations have been reported including, *Hyoscyamus muticus* L. (Egyptian Henbane)^11^. *H. muticus* L., a shrub thriving in Egypt’s sandy regions, possess notable pharmacological properties which may arise either intrinsic bioactive compounds or from its associated endophytic fungi^63^. In the present study, fungal endophytes and their secondary metabolites were isolated from aerial parts of *H. muticus*. Eight isolates were obtained and identified morphologically, among which *Fusarium equiseti* was further confirmed via morphological traits, molecular characterization and ITS of rDNA sequencing. The isolate of *F. equiseti* showed 99.61%–100% identity with previously reported strains. The presence of *F. equiseti* aligns with regional biodiversity studies; for instance^64^, isolated ten endophytic fungi from *H*. *muticus* L. grown in Wadi-Elnatrun Valley, whereas Abdel-Motaal *et al*. ^65^ demonstrated a total of 44 endophytes, with *Aspergillus fumigatus* being the most prevalent species across *H*. *muticus* tissues. *Fusarium* species are widely recognized as a prolific producer of diverse secondary metabolites (SMs) with broad biological activities. However, antimicrobial SMs from *Fusarium* are relatively under documented, despite a report by Xu *et al*.^66^ detailing 185 antimicrobial natural products from *Fusarium* strains by 2022. To fully explore the metabolic potential of the isolate, fermented culture was extracted with ethyl acetate which is widely reported as an efficient solvent for fungal metabolites extraction^67^. Given that endophytes are often a strong source of antibacterial compounds^68^. The resulting ethyl acetate extract was then subjected to comprehensive in vitro screening.

Prior to bioactivity testing, secondary metabolites ethyl acetate extract *of F. equiseti* (FE) was analyzed using GC–MS analysis to identify and profile the chemical constituents responsible for the observed biological activities. The analysis successfully revealed the presence of 17 bioactive compounds, primarily belonging to fatty acid class, known to possess antioxidant, and anti-microbial activities. Key findings included the identification of hexadecenoic acid (palmitic acid), a major component known for its antioxidant and anti-inflammatory activities, and octadecanoid acid (stearic acid) which primarily contributes to the antioxidant capacity^44,45,69^. Furthermore, fatty acids derivatives such as n-hexadecanoic acid and 9,12-octadecadienoic acid methyl ester (E, E), were detected, compounds often linked to enhanced antibacterial activity against various strains^56^. Notably, Linoleic acid, one of the detected components, is known to inhibit *S*. *aureus*, including multidrug-resistant strains^66^.

Concurrently, the FE extract was screened for antioxidant, antimicrobial, anti-inflammatory, and cytotoxic activities. The cumulative concentration of phenolic compounds accounts for the moderate antioxidant activity (IC50 = 145.91 µg/ml) observed in the extract. *F. equiseti* BwKpRt-1 showed a strong antibacterial and moderate antioxidant activity (IC50 = 57.36 µg/ml)^70^. The *F. equiseti* extract also exhibited significant antioxidant activity, correlated with its total polyphenol contents as supported by prior studies on *F.* and *Alternaria* spp.^71,72^. Consistent with literature on Fusarium derived metabolites, the extract exhibited strong antibacterial activity against *B. subtilis*, *S. aureus*, *E. coli*, *P. aeruginosa*, *C. albicans*, and *A. fumigatus*. This broad-spectrum activity is likely linked to specific metabolites classes, notably phenolic compounds, fatty acids, and diketopiperazines^65,73^. Previous studies have also documented antibacterial ^74^ and cytotoxic^75,76^ activities from *Fusarium* metabolites including *F. incarnatum–equiseti* complex^77^*.*

Crucially, cytotoxic assessment revealed a desirable selective toxicity (SI) toward HeLa cancer cells while sparing normal WI38 cells. The SI reflects the differential activity of extract; a higher SI value indicates greater selectivity. Conversely, an SI value below 2 suggests that the pure compound exhibits general toxicity ^78^. This selective toxicity towards cancer cells is a property that is increasingly regarded after in the development of new anticancer treatments, suggesting that *F. equiseti* holds significant promise as a source for developing pharmacologically relevant compounds.

To evaluate real world biomedical relevance, an *in vivo* hepatorenal toxicity protection model was established using cadmium chloride (CdCl_2_), a well-documented environmental pollutant with a biological half-life of 20–30 years in human. Due to its extremely poor elimination rate, cadmium accumulates in soft tissues- primarily liver and kidneys via systemic circulation and classified as one of the most hazardous metals to human health inducing hepatic, renal and neurological injury^2^. In this context, the present work evaluated whether secondary metabolites derived from the endophytic fungus *Fusarium equiseti* (FE) could mitigate oxidative tissue damage caused by CdCl_2_.

Organ toxicity assessment in xenobiotic research has been shown to be significantly impacted by both body weight and organ weight. In most cases, the influence of xenobiotics or Cd on weight loss or increase is reflected in the results of the toxicity study^79^. Along with the control group, rats given 100 mg FE/kg b.w. and 3 mg CdCl_2_/kg b.w. also gained weight over time. This means that neither FE nor the CdCl_2_ levels studied exhibited any acute toxicity. In addition, the experimental rats (those given CdCl_2_ as well as those given FE alone or in combination) showed no changes in relative organ weight.

To assess the level of toxicity of medicinal compounds, including extracts of plants, researchers analyze hematological indicators^80^, making changes in blood parameters are good signs of harmful medications, chemicals, heavy metals, etc. Aside from a small rise in the total count of white blood cell (WBC) (*P* < 0.05)^81^ and lymphocyte count, CdCl_2_ treatment did not significantly alter blood parameters in this investigation. The immunological response to foreign chemicals is mediated by lymphocytes, which are dynamic cells^82^. The presence of systemic inflammation is indicated by this rise^83^. Aligning with findings by El-Demerdash *et al*.^81^, that animal given CdCl_2_ had higher white blood cell counts. a possible indication of immune system activation. One essential way to measure blood glucose balance is fasting blood glucose (FBG). When compared to the control group, rats given CdCl_2_ had higher blood FBG levels.

Serum liver function testing provides insight into liver health. The amounts of albumin (ALB) and total protein determine the liver’s functioning, whereas the integrity of the liver’s cells is defined by the enzymes ALT and AST^84^. Serum levels of AST and ALT may be raised in response to liver injury since these enzymes are primarily generated by hepatocytes^70^. Although ALT is most reliable biomarker for detecting liver injury or hepatotoxicity, it is also prevalent in the heart, kidneys, skeletal muscle, and testes^85^. Consistent with earlier results showing that CdCl_2_ harms the liver and is a useful indicator of its function, our investigation found that liver enzyme levels were elevated in rats given CdCl_2_ alone^86^. High levels of transaminases in the blood indicate that there is significant damage to the liver cell membranes, which allows transaminases to seep into circulation^87^. Reduced levels of ALB and bilirubin, which are indicators of impaired synthesis function in the liver, further support the evidence of hepatic damage and illness^88^.

Blood creatinine is a critical indicator of renal function and glomerular filtration rate, with normal baseline levels in rats typically falling between 0.2–0.8 mg/dL^89^. Our results verify that rats exposed to CdCl_2_ had considerably elevated blood creatinine levels and suffered from severe kidney injury. In laying hens exposed to CdCl_2_, Zhu *et al*.^90^ found that blood creatinine and urea levels were similarly dramatically raised. Cd induced disruption of lipid homeostasis in this study is consistent with its characterization as a systematic metabolic disruptor mediated primarily through redox imbalance. The dyslipidemia recorded in CdCl_2_- exposed rats aligns with prior evidence suggesting that Cd promotes oxidative stress driven modulation of lipid synthesis and degradation pathways. Such disturbances are generally attributed to an overabundance of reactive oxygen species (ROS), which in turn set off a cascade of negative biological responses that manifest as pathophysiological processes, abnormal cellular function, and dyslipidemia^2^.

The key mechanisms by which cadmium-induced hepato-renal toxicity is recognized to include cellular inflammation, lipid peroxidation, ROS formation, and a reduction in antioxidant defense systems such catalase (CAT), reduced glutathione (GSH), glutathione peroxidase (GPx), and superoxide dismutase (SOD)^91^. Excessive formation of free radicals is the primary cause of glutathione depletion, which is in line with earlier findings that indicated the metal interacts with SH groups of glutathione, a critical component of the intracellular antioxidant defense system, leading to an oxidative stress state. This is paralleled by an elevation in MDA levels when GSH levels fall under Cd exposure conditions^2^. Metabolic dysfunction and instability of cellular integrity are consequences of ROS like superoxide, hydroxyl radicals, and hydrogen peroxide which primarily impact lipids, carbohydrates, and proteins. Moreover, MDA is a key indicator in the process of lipid peroxidation. It damages parenchymal cells, disrupts many biomolecules (including DNA and acetaldehyde), and encourages the formation of advanced end products of glycation, which play essential for cellular integrity^86^. Notably, bioactive compounds derived from endophytic fungus have demonstrated potential in mitigating ROS levels. In the current work, FE reduced MDA content (p < 0.001), indicating antioxidant activity. These results align with prior reports on the antioxidant capacity of fungi isolated from *Bauhinia variegata* leaves^92^.

Histological analysis corroborated these biochemical alterations, revealing a significant pathological alteration in liver and kidney, most of which may attributed to oxidative injury^86^. The observed hepatic lesions coincide with elevated serum transaminases, whereas renal pathological alterations were reflected by elevated serum creatinine levels. These findings align with the established view of Cd as a cumulative nephrotoxicant^18,20^. Remarkably, pre-treatment with FE conferred substantial protection against CdCl_2_ biochemical and histological lesions. FE extract administration restored lipid parameters, attenuated oxidative stress indicators, and re-established redox homeostasis, suggesting potent antioxidant capabilities.

The normalization of blood indicators including creatinine, ALT, and AST, imply both hepato- and nephroprotective efficacy. These observations are congruent with prior study demonstrating reno-protective effects of *Phomopsis* sp. fungal extract which modulated creatinine, ALT, and AST within physiological limits^19^. Comparable histological improvements following co-administration of fungal metabolites have been documented previously. Saleh *et al*.^17^ reported that concurrent treatment with mushroom derived extracts markedly attenuated tissue degeneration over time. Similarly, another study demonstrated that *F. equiseti* extract facilitated the restoration of hepatic and renal architecture in rats with hepatocellular carcinoma evidenced by less fatty infiltration and improved cellular organization.

The broad-spectrum biological activities exhibited by the FE extract can be mechanistically may attributed to its chemically diverse metabolite profile identified by GC–MS analysis, many of which have been previously reported to possess significant pharmacological activities^49,50,60–62^. The antioxidant activity of the extract is primarily mediated by fatty acids and their esters, such as palmitic acid, oleic acid, and their methyl esters, in addition to phenolic compounds, which collectively act as hydrogen- and electron-donating agents to neutralize ROS. These compounds also inhibit lipid peroxidation and enhance endogenous antioxidant defenses, including SOD and GSH, leading to reduced MDA levels, a key marker of oxidative stress^16,18,19,57,58^. Moreover, long-chain alcohols such as 1-eicosanol and 1-hexadecanol derivatives contribute to redox balance through membrane-associated antioxidant effects^60^. Furthermore, antimicrobial activity can be explained by the presence of lipophilic compounds such as fatty acids (palmitic and oleic acids), esters, and hydrocarbons (10-heneicosene, 1-docosene, and heptacos-1-ene), which are known to disrupt microbial cell membrane integrity, leading to increased permeability, leakage of intracellular components, and eventual cell death^16,49,54,55^. Additionally, bis(2-ethylhexyl) phthalate and unsaturated fatty acids have been reported to exhibit potent antibacterial and antifungal activities through interference with microbial metabolic pathways^50^.

The anti-inflammatory activity observed in the HRBC membrane stabilization assay is likely mediated by fatty acids and phenolic derivatives, which stabilize biological membranes and inhibit the release of inflammatory mediators. These compounds are also known to modulate key inflammatory pathways, including cyclooxygenase and lipoxygenase, thereby reducing the production of pro-inflammatory mediators^16,17,58,61^. The cytoprotective effects, particularly against cadmium-induced hepatorenal toxicity, may be attributed to a synergistic multi-target mechanism involving attenuation of oxidative stress, stabilization of cellular membranes, modulation of inflammatory responses, and restoration of biochemical and histological integrity. Fatty acids such as oleic, palmitic, and linoleic acids, along with their esters and phenolic constituents, play a central role in scavenging ROS, preventing lipid peroxidation, and restoring antioxidant enzyme levels, which collectively contribute to improved liver and kidney function and tissue recovery^18,20,57^.

Importantly, the high proportion of esters (~ 67%) and fatty acids (~ 26.34%) in the extract further supports its potent bioactivity, as these compounds act synergistically to produce multi-modal biological effects. This synergism between fatty acids, esters, hydrocarbons, alcohols, and phenolic derivatives enhances the overall efficacy of the crude extract compared to individual isolated compounds, highlighting *F. equiseti* as a promising natural source of multifunctional therapeutic agents^32^.

### Limitations

Although the present findings demonstrate the promising biological potential of *Fusarium equiseti* metabolites, the reliance on an acute CdCl₂ intoxication model limits the direct extrapolation of these results to chronic environmental or occupational exposure scenarios. Therefore, future studies should prioritize elucidating the molecular signaling pathways underlying CdCl₂-induced toxicity, alongside bioassay-guided fractionation, compound isolation, and structural characterization. Such approaches are essential to identify the specific bioactive constituents and to validate their safety and protective relevance at the molecular level.

## Conclusion

The secondary metabolites extracted from *Fusarium equiseti* demonstrated multiple biological activities, including antimicrobial, cytoprotective, and antioxidant effects. The *in vivo* data indicates that the FE extract exerts protective effects against CdCl_2_- induced hepatorenal toxicity. The mitigation of biochemical disruptions, restoration of antioxidant capacity, and reversal of histological damage support its protective potential. The presence of organic acids, phenolic compounds, and other bioactive components, likely underlies these protective mechanisms through scavenging of free radicals and reinforcement of endogenous antioxidant defense systems. These results not only validate the antioxidative efficacy of FE extract but also highlight its potential as a candidate for further investigation in hepato and reno-protective agents against heavy metals toxicity.

## Supplementary Information

Supplementary Information.

## Data Availability

The data of fungal strain Fusarium equiseti AUMC 15,585 with GenBank accession no. OP694170, you may find the datasets created and/or used in this work in the NCBI repository at: https://www.ncbi.nlm.nih.gov/nuccore/OP694170.1?report=genbank

## Abbreviations

BLAST: Basic local alignment search tool
CdCl_2_: Cadmium chloride
*F. equiseti*: *Fusarium equiseti*
FE: Fusarium secondary metabolites extract
*H*. *muticus*: *Hyoscyamus muticus*
PDA: Potato dextrose agar
