# Supplementary material for: Bioactive metabolites from endophytic Fusarium equiseti isolated from Hyoscyamus muticus mitigate cadmium toxicity in biological models
Source: Sci Rep. 2026 Jun 30;16:19931. doi: 10.1038/s41598-026-59146-x (PMC13319267; doi:10.1038/s41598-026-59146-x)
Supplement: Supplementary file 1 — Supplementary Information. [file 41598_2026_59146_MOESM1_ESM.docx]

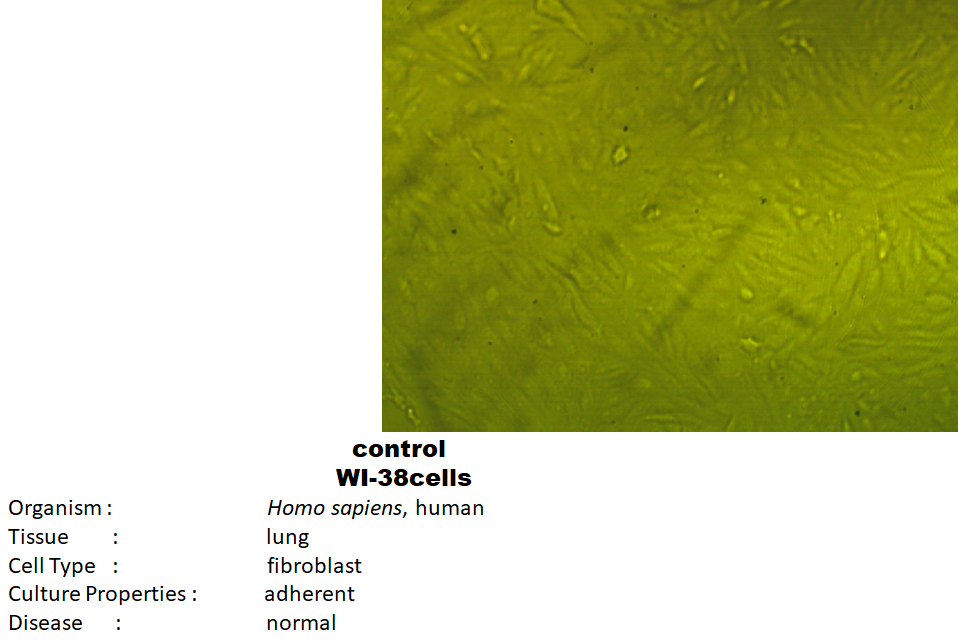

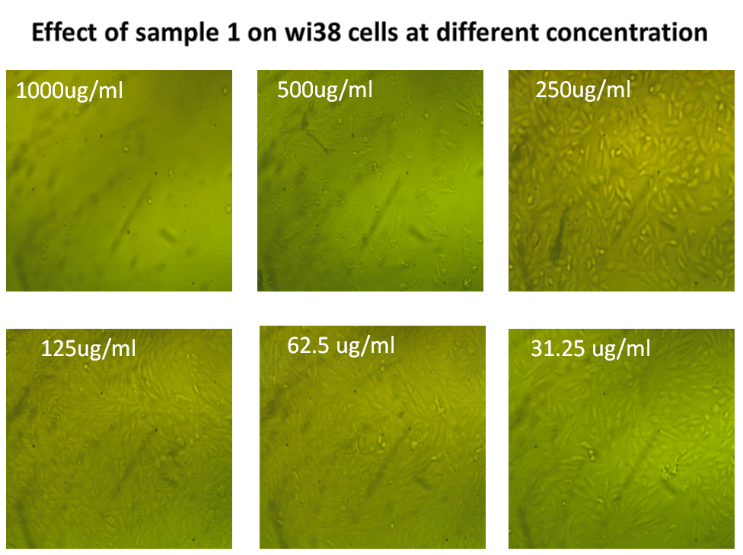


**Supplementary** Figure 1. Morphological changes in Wi38 cells following treatment with *Fusarium equiseti* ethyl acetate extract. (A) Untreated control cells showing normal morphology. (B) Dose-dependent cytotoxic effects at varying extract concentrations (1000–31.25 µg/mL), with higher doses leading to cell shrinkage, detachment, and reduced confluency.


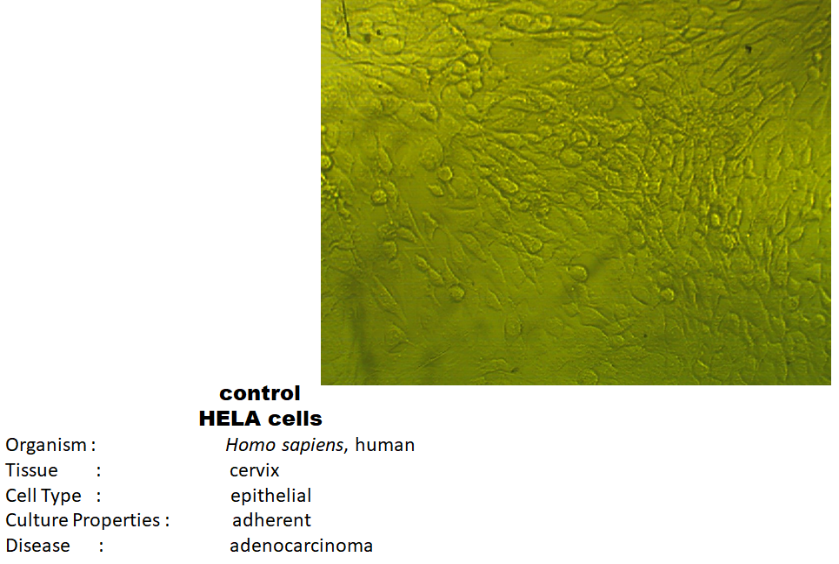

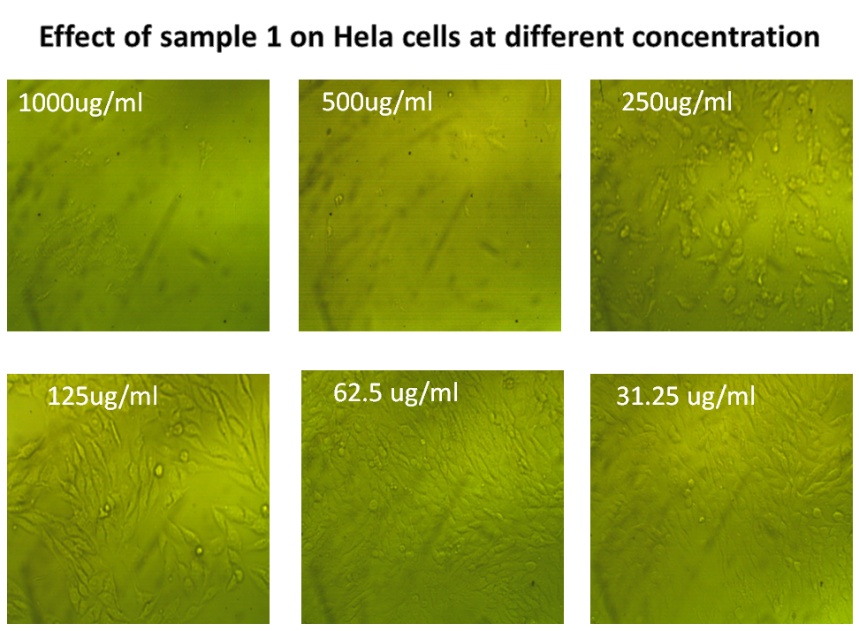


**Supplementary** Figure 2. Morphological changes in HeLa cells following treatment with *Fusarium equiseti* ethyl acetate. (A) Untreated control cells showing normal morphology. (B) Dose-dependent cytotoxic effects at varying extract concentrations (1000–31.25 µg/mL), with higher doses leading to cell shrinkage, detachment, and reduced confluency.

.


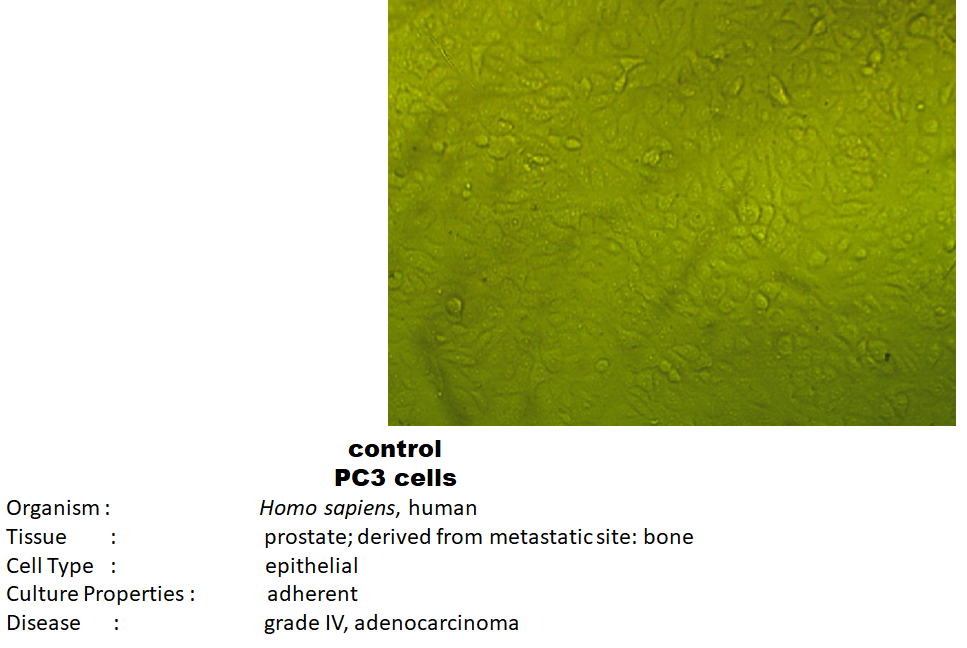

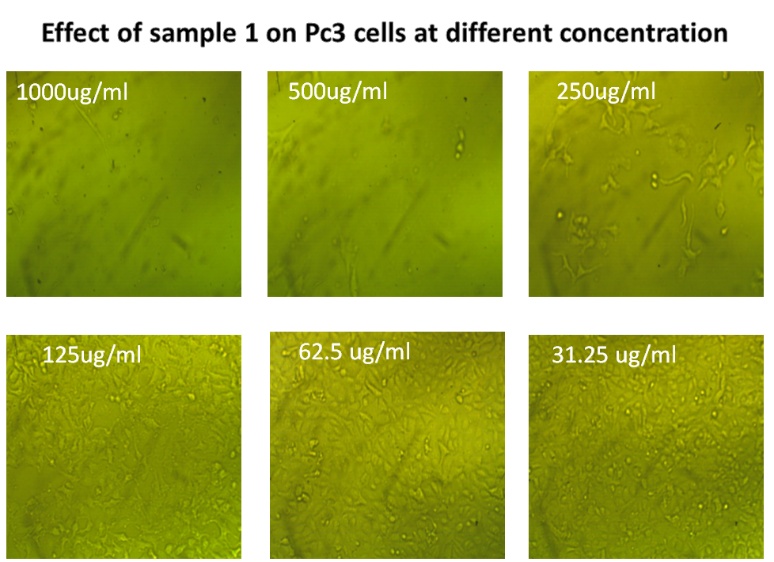


**Supplementary** Figure 3. Morphological changes in PC3 cells following treatment with *Fusarium equiseti* ethyl acetate extract. (A) Untreated control cells showing normal morphology. (B) Dose-dependent cytotoxic effects at varying extract concentrations (1000–31.25 µg/mL), with higher doses leading to cell shrinkage, detachment, and reduced confluency.

**Supplementary Table 1. *In silico* ADME analysis of tested phytochemicals.**

| **Descriptors** | **Bis(2-ethylhexyl) phthalate** | **9,12-Octadecadienoic acid (Z,Z)** | **Oleic Acid (9-Octadecenoic acid, Z)** | **n-Hexadecanoic acid** |
| --- | --- | --- | --- | --- |
| SMILE | CCCCC(CC)COC(=O)C1=CC=CC=C1C(=O)OCC(CC)CCCC | CCCCC/C=C\C/C=C\CCCCCCCC(=O)O | CCCCCCCC/C=C\CCCCCCCC(=O)O | CCCCCCCCCCCCCCCC(=O)O |
| **Physicochemical properties** | | | | |
| Number of heavy atoms | 28 | 20 | 20 | 18 |
| Number of aromatics  heavy atoms | 6 | 0 | 0 | 0 |
| Fraction Csp3 | 0.67 | 0.72 | 0.83 | 0.94 |
| Number of rotatable  Bonds | 16 | 14 | 15 | 14 |
| Number of H-bond  acceptors | 4 | 2 | 2 | 2 |
| Number of H-bond donors | 0 | 1 | 1 | 1 |
| Molar refractivity | 116.30 | 89.46 | 89.94 | 80.80 |
| Topological polar surface  area (Å2) | 52.60 | 37.30 | 37.30 | 37.30 |
| **Lipophilicity** | | | | |
| Log Po/w (iLOGP) | 4.77 | 0 | 4.01 | 3.85 |
| Log Po/w (XLOGP3) | 7.45 | 0 | 7.64 | 7.17 |
| Log Po/w (WLOGP) | 6.43 | 5.88 | 6.11 | 5.55 |
| Log Po/w (MLOGP) | 5.24 | 0 | 4.57 | 4.19 |
| Log Po/w (SILICOS-IT) | 6.98 | 0 | 5.95 | 5.25 |
| Consensus log Po/w | 6.17 | 0 | 5.65 | 5.20 |
| **Water solubility** | | | | |
| Log S (ESOL) | -6.06 | 0 | -5.41 | -5.02 |
| Solubility (mg/ml) | 3.42e-04 | - | 1.09e-03 | 2.43e-03 |
| Class | Poorly soluble | - | Moderately soluble | Moderately soluble |
| Log S (Ali) | -8.39 | - | -8.26 | -7.77 |
| Ali Solubility (mg/m | 1.60e-06 | - | 1.54e-06 | 4.31e-06 |
| Class | Poorly soluble | - | Poorly soluble | Poorly soluble |
| Log S (SILICOS-IT) | -7.40 | - | -5.39 | -5.31 |
| Solubility | 1.56e-05 | - | 1.14e-03 | 1.25e-03 |
| Class | Poorly soluble | - | Moderately soluble | Moderately soluble |
| **Pharmacokinetics** | | | | |
| GI absorption | High | - | High | High |
| BBB permeant | No | - | No | Yes |
| P-gp substrate | Yes | - | No | No |
| CYP1A2 inhibitor | No | - | Yes | Yes |
| CYP2C19 inhibitor | No | - | No | No |
| CYP2C9 inhibitor | Yes | - | Yes | Yes |
| CYP2D6 inhibitor | No | - | No | No |
| CYP3A4 inhibitor | Yes | - | No | No |
| Log Kp (skin permeation)  (cm/s) | -3.39 | - | -2.60 | -2.77 |
| **Drug-likeness** | | | | |
| Lipinski | 1 | 0 | 1 | 1 |
| Ghose | 1 | - | 1 | 0 |
| Veber | 1 | - | 1 | 1 |
| Egan | 1 | - | 1 | 0 |
| Muegge | 2 | - | 1 | 1 |
| Bioavailability score | 0.55 | - | 0.85 | 0.85 |
| **Medicinal chemistry** | | | | |
| PAINS | 0 | 0 | 0 | 0 |
| Brenk | 1 | 0 | 1 | 0 |
| Lead likeness | 3 | - | 2 | 2 |
| Synthetic accessibility | 4.12 | - | 3.07 | 2.31 |
